# Deep learning and high-resolution magnetic resonance vascular wall imaging: current challenges and future perspectives

**DOI:** 10.3389/fneur.2026.1731783

**Published:** 2026-02-20

**Authors:** Zhiming Cui, Jibo Hu, Huiqing Zhang

**Affiliations:** Department of Radiology, The Fourth Affiliated Hospital of School of Medicine and International School of Medicine, International Institutes of Medicine, Zhejiang University, Yiwu, China

**Keywords:** artificial intelligence, cerebrovascular diseases, deep learning, high-resolution magnetic resonance, vascular wall imaging

## Abstract

High-resolution magnetic resonance vessel wall imaging (HR-VWI) is an advanced MR imaging technique that can directly visualize intracranial vessel walls and detect subtle pathological changes. HR-VWI can improve diagnostic confidence, help differentiate intracranial vascular diseases, and assist in patient risk stratification and prognosis. However, HR-VWI relies heavily on operator experience and is therefore unreliable in inexperienced hands. Deep learning (DL) is considered a leading artificial intelligence tool in image analysis. DL algorithms excel at image recognition by leveraging multimodal data, making them valuable in medical imaging. Recently, a growing number of studies have proposed the use of DL models as tools to support radiologists and overcome the inherent challenges of MR imaging. DL has numerous clinical applications in cerebral angiography, including the identification of intracranial aneurysms, arteriovenous malformations, arteriosclerosis, and moyamoya disease. This article comprehensively reviews the fundamentals of DL and its applications in HR-VWI, with a particular focus on its clinical applications in assessing various intracranial vascular lesions. DL-assisted HR-VWI has the potential to become an important ancillary diagnostic tool for cerebrovascular diseases.

## 1 Introduction

Cerebrovascular disease (CVD) represents one of the leading etiological factors contributing to mortality and long-term disability on a global scale. According to data derived from the Global Burden of Disease (GBD) Study 2021, stroke ranks as the third most prevalent cause of death globally, accounting for 10.7% of total global mortality. Specifically, the global incidence of stroke stands at 93.8 million cases, with 11.9 million new incident cases reported annually. Ischemic stroke accounts for 65.3% of all stroke cases (1). Although the age-standardized mortality rate (ASMR) of ischemic stroke has exhibited a downward trend, the absolute incidence and disease impact of this condition remain persistently high, which is attributed primarily to factors such as the aging of the global population (2). Notably, the distribution of stroke burden varies significantly across different populations. Southern sub-Saharan Africa had the highest age-standardized rates of ischemic stroke incidence (ASPRs), and Eastern Europe had the highest incidence age-standardized rates of ischemic stroke incidence (ASIRs). The greatest age-standardized rates of ischemic stroke mortality (ASDR) and age-standardized DALY rates were observed in Eastern Europe, Central Asia, North Africa, and the Middle East (3). Intracranial atherosclerotic disease (ICAD) and intracranial artery stenosis (ICAS) are leading causes of ischemic stroke worldwide. Significant racial and ethnic disparities are widely accepted to affect the prevalence of ICAD and ICAS. According to epidemiological studies, 30 to 70% of acute ischemic strokes (AISs) in Asian, Hispanic, and Black patients are caused by ICAS. In contrast, ICAS contributes to AIS in only 5 to 10% of white patients (4). Other intracranial vascular pathologies, including arterial dissection, vasculitis, and moyamoya disease (MMD), can individually or collectively lead to cerebral ischemia, transient ischemic attacks, or hemorrhagic complications. These conditions subsequently determine the patient’s treatment strategy and prognostic pathway (5, 6). Therefore, precise etiological diagnosis and risk assessment of intracranial vascular pathologies are crucial for developing individualized treatment strategies and improving patient outcomes.

Currently, the clinical evaluation of intracranial vascular etiology largely relies on noninvasive vascular imaging techniques such as magnetic resonance angiography (MRA), computed tomography angiography (CTA), and digital subtraction angiography (DSA). These methods primarily assess morphological changes, including the degree of vascular stenosis or occlusion or the presence of aneurysms (7–9). These techniques are highly valuable for assessing hemodynamic impairment. For example, CTA combined with CT perfusion (CTP) has demonstrated strong diagnostic performance in detecting distal medium vessel occlusions (7). However, a fundamental limitation lies in their inability to directly visualize the pathological characteristics of the vessel wall itself (10–12). Critical information about the vessel wall—such as atherosclerotic plaque composition, intramural hematoma, and inflammatory activity—is essential for determining the stability, activity, and etiology of the lesion. However, such information is often difficult to capture with conventional vascular imaging (9, 12, 13).

The advent of high-resolution magnetic resonance vessel wall imaging (HR-VWI) has provided a novel perspective for in-depth elucidation of the pathophysiological alterations in the intracranial vasculature. By transcending the limitations of luminal assessment, HR-VWI enables direct visualization of vascular wall morphology, thickness, signal characteristics, and contrast enhancement patterns through optimized sequence design and blood flow signal suppression. This ability provides critical evidence for etiological differentiation, pathological stratification, and risk assessment of lesions (9, 14). Radiomics analyses based on HR-VWI can be used to evaluate plaque enhancement—an imaging surrogate for inflammatory activity (15). For example, Chen et al. enrolled 100 patients (aged 18--80 years) with middle cerebral artery (MCA) plaques in China. Compared with traditional plaque characteristic analysis, which had AUCs of 0.744 and 0.700 in the training and test sets, the radiomics and combined models presented increased AUCs in identifying culprit plaques: 0.860 and 0.896 in the training sets and 0.795 and 0.833 in the test sets, respectively (16). Guo and colleagues enrolled 601 patients from China (mean age, 53.5; male/female, 66.6%/33.4%) with intracranial atherosclerotic disease (ICAD) who underwent percutaneous transluminal angioplasty and stenting (PTAS). The patients were divided into cohorts, including a training cohort (*n* = 336), a validation cohort (*n* = 144), and a test cohort (*n* = 121). The integrated model had AUCs of 0.93 in the training cohort, 0.87 in the validation cohort, and 0.87 in the test cohort in predicting the risk of periprocedural complications associated with endovascular therapy (17). In a cohort of 20 patients (15 men, 5 women; mean age = 62.85 ± 10.56 years) with chronic internal carotid artery occlusion, HR-VWI has added value over DSA in evaluating intraluminal thrombi, which can help select candidates for revascularization (18). However, the widespread clinical adoption and application of HR-VWI face several challenges: (1) manual segmentation of the vessel wall and plaque requires continuous training of personnel and is labor intensive; (2) manual segmentation is time-consuming, which usually takes a trained expert more than 30 min to analyze images of one patient; and (3) the low contrast between the vessel wall and the surrounding tissues often affects the accuracy of segmentation. This work depends heavily on the knowledge and experience of experts.

In recent years, deep learning (DL), a core technology within the realm of artificial intelligence (AI), has undergone rapid advancement and achieved extensive application in medical image analysis. DL offers a novel avenue to address the aforementioned challenges. As a subfield of machine learning, DL enables the automatic extraction of complex images from large-scale datasets through the construction of deep neural network models, consequently demonstrating exceptional performance in tasks such as image segmentation, classification, detection, and generation (19, 20). DL has been successfully applied to the automatic classification of stroke subtypes on the basis of diffusion-weighted imaging (DWI) (21), the identification and staging of infarcts via noncontrast computed tomography (NCCT) (22, 23), and the prediction of stroke outcomes via multimodal CTP parameters (24). For example, Zhang et al. enrolled 65 pediatric patients (36 males and 29 females, mean age = 6.38 ± 4.77 years) who underwent epilepsy surgery in South China. High-resolution brain MRI structural data were collected. The DL model, which was named 3D full-resolution nnU-Net, exhibited good performance in identifying focal cortical dysplasia (FCD) at a sensitivity of 0.73. The mean Dice–Sørensen coefficient (DSC) was 0.57 for automatic segmentation (25).

**Figure 1:**
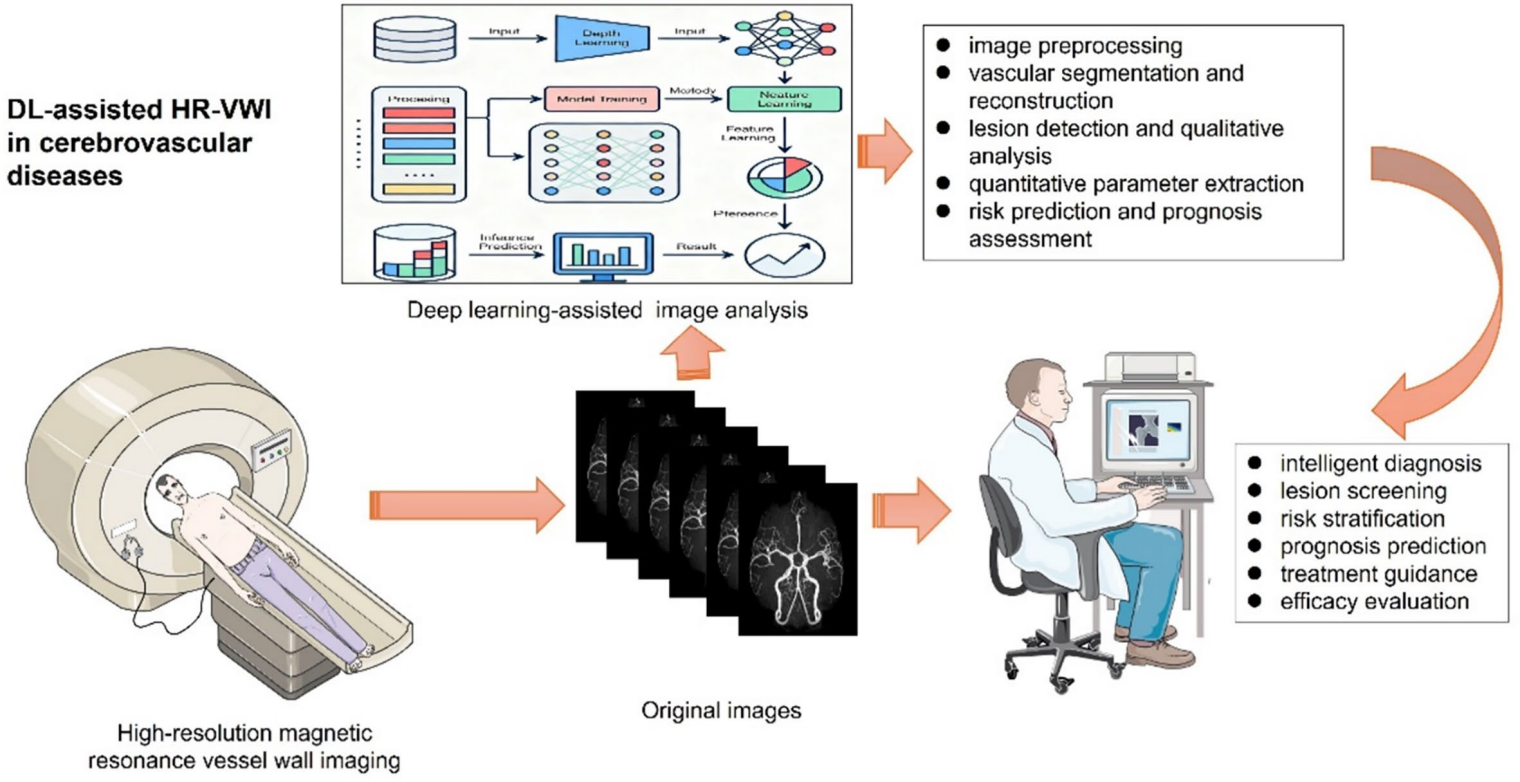
The workflow diagram of DL-assisted HR-VWI in cerebrovascular diseases. High-resolution magnetic resonance vessel wall imaging (HR-VWI) images were acquired and analyzed via different DL models, which generally include (1) image preprocessing, (2) vascular segmentation and reconstruction, (3) lesion detection and qualitative analysis, (4) quantitative parameter extraction, and (5) risk prediction and prognosis assessment. DL-assisted HR-VWI may help radiologists or clinicians with (1) intelligent diagnosis, (2) lesion screening, (3) risk stratification, (4) prognosis prediction, (5) treatment guidance, and (6) efficacy evaluation.

The application of DL technology to HR-VWI analysis holds the promise of enabling automatic and precise segmentation of vascular wall boundaries, objective and quantitative extraction of plaque features, and intelligent assessment of lesion nature, activity, and prognosis on the basis of multiparametric medical images. For example, Chandrashekar et al. enrolled 75 patients with paired noncontrast and contrast-enhanced CT images. Compared with CT angiograms, the attention-based U-Net achieved better performance in extracting both the inner (blood flow) lumen and the wall structure of the aortic aneurysm, thus allowing for accurate and efficient extraction of the morphological and pathological features of the entire aortic volume (26). DL-assisted advancements in HR-VWI image analysis substantially increase the degree of automation, quantitative reliability, reproducibility, and clinical diagnostic efficiency (Figure 1). The present review aims to systematically summarize the research progress and clinical potential of DL integrated with HR-VWI for various intracranial vascular lesions, such as ICAD, intracranial aneurysm (IA), etiological differentiation of ischemic stroke, MMD, and arteriovenous malformation (AVM). Additionally, this study aims to analyze the current challenges and limitations and provide a perspective on future development directions, with the objective of offering insights to facilitate the advancement of precision medicine in cerebrovascular diseases.

## 2 Application of DL in intracranial vascular lesions

### 2.1 Application of traditional machine learning algorithms

Traditional machine learning (ML) methods have long been the main representatives of early exploration and interpretable methods in medical image analysis. These algorithms typically rely on manually designed and carefully extracted features, followed by the use of classifiers or regression models to identify patterns, perform classification, or predict outcomes in medical imaging, including vascular imaging (27–29). Common algorithms include K-nearest neighbor (KNN), linear discriminant analysis (LDA), support vector machine (SVM), random forest (RF), multilayer perceptron (MLP), and various clustering algorithms, among others. These methods often exhibit good performance and interpretability when dealing with datasets with relatively low dimensions and clear-meaning features.

Within the domain of cerebrovascular imaging analysis, a pivotal application direction of traditional ML lies in the differential diagnosis of lesions or risk stratification depending on imaging features. Typically, such studies follow a standardized workflow: (1) a large set of quantitative features is extracted from imaging data; (2) feature selection is performed to filter out the most discriminative feature subset; and (3) the selected subset is fed into a classifier for model construction. A key advantage of this approach lies in its ability to unravel complex feature combinations that are imperceptible to radiologists’ eyes. Zhao et al. conducted a multicenter study in South China that included a total of 645 patients with acute ischemic stroke or transient ischemic attack (232 patients were symptomatic, and 413 patients were asymptomatic). Among them, 317 patients (mean age: 70.20 ± 9.14 years; 159 women and 158 men) were included in the internal training set, and the other 169 patients (mean age: 70.71 ± 9.39 years; 85 women and 84 men) and 159 patients (mean age: 65.42 ± 10.41 years; 90 women and 69 men) were included in two external validation sets. A 256-slice CT scanner was used to perform the CTA scans. This study leveraged the radiomic features of perivascular adipose tissue (PVAT) surrounding the carotid artery, integrated with the training of multiple classifiers—including KNN, SVM, logistic regression (LR), RF, LDA, multinomial naive Bayes (multinomialNB), and extreme gradient boosting (XGBoost). By integrating the radiomic score (Rad score), this combined model performed excellently in predicting symptomatic carotid plaques (30). SVMs are frequently employed for binary classification tasks, such as distinguishing electroencephalographic signals between poststroke mild cognitive impairment and vascular dementia (27), primarily because of their robust performance on small-sample and high-dimensional datasets. In contrast, RF is commonly used to predict the risk of future cerebrovascular events in patients with carotid artery stenosis because it enables feature importance evaluation and is relatively insensitive to overfitting (31).

In addition to supervised learning tasks, unsupervised clustering algorithms play a pivotal role in exploratory analysis of disease subtypes. For example, in a notable study, clustering methods were employed to investigate the correlation between necroptosis of cells and immune responses in MMD, with the primary objective of identifying potential biological subtypes (32). This exploratory approach circumvents the need for predefined labels, enabling the discovery of latent disease heterogeneity that may be overlooked by hypothesis-driven analyses. Furthermore, in scenarios where data annotation is challenging—often characterized by labor-intensive processes or limited availability of expert-labeled samples—machine learning methodologies have been leveraged to generate synthetic imaging data for training set augmentation. A typical application is the generation of cerebral microbleed images for classifier training, particularly in contexts where a definitive gold standard for annotation is absent (33). This data augmentation strategy not only mitigates the constraint of insufficient labeled data but also enhances the robustness of subsequent models by introducing diverse synthetic samples that mimic real-world imaging variations. In summary, traditional ML algorithms have provided preliminary solutions for the automated analysis of intracranial vascular lesions and have demonstrated their feasibility in specific tasks. However, their inherent limitations—including heavy reliance on manual feature engineering, high sensitivity to the quality of image preprocessing, and inadequate generalizability across different clinical centers—have propelled research efforts toward DL techniques. These DL approaches are distinguished by their capacity to automatically learn hierarchical feature representations from raw data, thereby addressing the key shortcomings of traditional ML in medical imaging analysis.

### 2.2 Application and development of DL

As a pivotal subfield of ML, DL simulates the hierarchical information processing mechanism of the human brain by constructing neural networks with multiple hidden layers, thereby demonstrating immense potential in medical imaging analysis (34, 35). Unlike traditional ML methods that rely on handcrafted features, DL possesses the inherent capability to automatically learn multilevel feature representations directly from raw data—a characteristic that renders it particularly suited for analyzing complex imaging datasets such as HR-VWI. The convolutional neural network (CNN) stands out as one of the core DL architectures for processing imaging data (34, 36). Typical CNN models, such as AlexNet, VGG, GoogleLeNet, and ResNet, were developed primarily for image classification. Through the integration of convolutional layers, pooling layers, and fully connected layers, CNNs can capture local features and spatial hierarchical structures within images. These CNN models are heavily reliant on both the size of the training dataset and the accuracy of image annotations. In many medical image analysis tasks—particularly in 3D scenarios—it is often difficult to assemble a sufficiently large and high-quality training dataset because of challenges in data collection and annotation (37).

**Figure 2:**
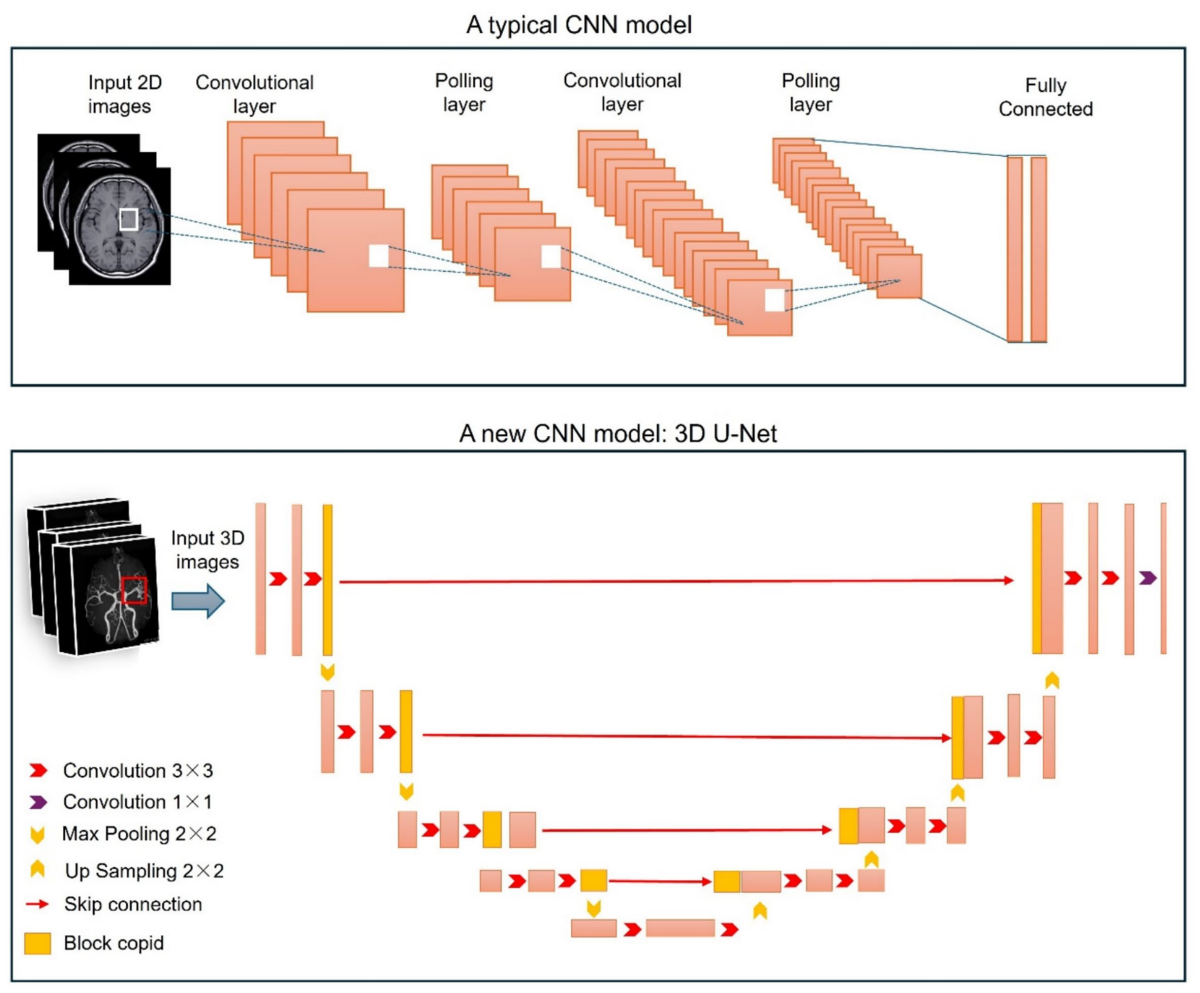
The network architecture of a typical CNN model and a new CNN model for HR-VWI. The typical CNN (upper panel) directly takes an image as input, then transforms it via convolutional layers, pooling layers, and fully connected layers, and finally outputs a class-based likelihood of that image (125). One example of a new CNN (lower panel): this 3D U-Net model employs a U-Net structure enhanced with shortcut connections within each convolutional block. This model demonstrated superior segmentation performance in comparison with an equivalent 2D implementation (126).

U-Net is specifically designed for medical image segmentation, and it significantly differs from traditional CNNs in terms of the task characteristics of vessel segmentation, network structure, training methods, and segmentation performance (Figure 2). Compared with the traditional CNNs’ asymmetric structure, where the decoder is simple (only upsampling + convolution) and lacks a dedicated fusion structure, U-Net adopts a symmetric encoder–decoder plus skip connection structure. In traditional CNNs, the decoder only performs upsampling of images, losing a large number of low-dimensional detail features. The max pooling process continuously loses spatial detail information (such as pixel coordinates and edge textures) and retains only high-level semantic features (such as “vessel area”), resulting in missed segmentation of small vessels, blurred vessel edges, and even misclassification of similar tissues around the vessels as vessels (38). U-Net’s dense skip connections are the core advantage. Low-dimensional details extracted by the encoder (such as the thin tubular edges of vessels) are directly transmitted to the corresponding layers in the decoder and fused with the global semantic features restored by upsampling. This not only ensures the spatial localization accuracy (clear edges) of the segmentation result but also enables accurate recognition of small vessels/fine branches, perfectly adapting to the fine-grained structure of vessels. Each layer of the decoder contains convolution operations to further refine the fused features, remove background noise (such as surrounding tissues and image artifacts), and enhance the saliency of vessel features (39, 40). U-Net’s output is at the same resolution as the input, whereas traditional CNNs often require interpolation or upscaling of the output after modification, leading to further loss of pixel accuracy, which is especially pronounced in the segmentation of fine structures such as vessels (41). In terms of training, traditional CNNs are highly dependent on large datasets and have weak generalizability, whereas U-Net is efficient in small sample training and is well adapted to the characteristics of medical imaging. U-Net’s elastic deformation data augmentation method applies nonrigid deformation tailored to the texture/structural characteristics of medical images, enabling the generation of many diverse training samples from a small number of annotated samples and greatly improving the model’s generalization ability (42). In terms of specific performance in vessel segmentation tasks, U-Net effectively captures weak features of small vessels/fine branches and suppresses background noise, and its segmentation results overwhelmingly surpass those of traditional CNNs in terms of completeness, precision, and robustness.

A major obstacle in medical imaging research is the limited availability of annotated data. Semisupervised learning (SSL) approaches have shown considerable promise in solving this issue in cerebrovascular segmentation. Osman and colleagues developed an SSL method with a dual-consistency approach that jointly pertains to pixel-image transformation, consistent equivariant and feature perturbation invariance. Two 3D time-of-flight (ToF) magnetic resonance angiography (MRA) datasets, including the IXI dataset (healthy subjects with approximately 600 TOF-MRI scans) and the TubeTK dataset (paired T1 and TOF-MRA images of 100 subjects), were used to evaluate the performance of this approach. The 3D UNet architecture was utilized as the teacher and student network, which was employed in collaboration to guide consistency regularization. Additionally, the pixel-level prediction performance was boosted by employing region-specific supervised loss only for the annotated input samples. The results showed that this method achieved a Dice similarity coefficient of 83.3% and an intersection-over-union of 71.5% on the IXI dataset (21). Such advances reduce the dependence on large, manually annotated datasets and help bridge the gap between research models and clinical implementation.

When working with time series medical imaging data, recurrent neural networks (RNNs) and their variant, long short-term memory (LSTM) networks, offer particular benefits (43, 44). A key strength of RNN-based models lies in their internal memory mechanism, which preserves historical information and allows them to capture temporal relationships in sequential data. For example, in analyzing dynamic functional MR images, convolutional recurrent neural networks (CRNNs) have been used to identify brain diseases by combining the spatial feature extraction ability of CNNs with the sequential modeling capacity of RNNs (43). This combined approach can identify spatial features in single fMRI images while also tracking changes over successive scans, leading to improved detection accuracy. Another common issue in longitudinal medical imaging—such as studies involving patient dropout or image artifacts—is incomplete data. Researchers have developed training methods to improve the ability of RNN and LSTM models to handle missing values. These improved models have been used, for example, in modeling the progression of Alzheimer’s disease, where longitudinal data often contain gaps (44). By making the models more robust to incomplete datasets, these methods support more reliable predictions of disease development. Overall, RNN- and LSTM-based approaches show promise for analyzing time-sensitive data in high-resolution vessel wall imaging (HR-VWI). Potential applications include dynamically enhanced HR-VWI, which tracks contrast agent movement over time, and multiple time point follow-up imaging, which is used to monitor changes in vascular lesions.

### 2.3 Role of the DL in the assessment of intracranial vascular lesions

DL-based algorithms are playing an increasingly pivotal role in the diagnosis and treatment workflow of intracranial vascular lesions, offering novel technical avenues to address bottlenecks inherent in traditional medical imaging analysis (Table 1). Spanning from image preprocessing to prognostic assessment, DL algorithms are gradually being integrated into various segments of clinical workflows, thereby providing robust support for the precise diagnosis and management of cerebrovascular diseases.

**Table 1:** Technical pathways and clinical applications of deep learning in intracranial vascular disease diagnosis and treatment.

Category	People	Conutry	Age	Gender	Application Direction	Representative Method	Technical Content	Ref.
Image Preprocessing	Healthy volunteers (*n* = 14)	South Korea	58.6 (40–67) years (men), 55.3 (33–65) years (women)	7 men, 7 women	Noise Reduction and Image Quality Enhancement	Self-Supervised and Unsupervised Deep Learning	Enhances image quality for compressed sensing MRI to improve signal-to-noise ratio	(45)
	10 healthy volunteers, 5 consecutive patients	N.A	N.A	N.A	Super-Resolution Reconstruction	3D Deep Learning Reconstruction	Improves 3D T1 SPACE vessel wall imaging quality and reduces scan time	(46)
	12 healthy subjects and 2 patients (coronary artery diseases)	Japan	Healthy subjects: mean age = 42.1 years; patients: 79 years/ 44 years	Healthy subjects: 6 males and 4 females; 2 patients: 1 male and 1 female	Motion Artifact Correction	K-Space Trajectory and Deep Learning Reconstruction	Reduces motion artifacts in coronary artery MR angiography	(47)
Vascular Segmentation and Reconstruction	66 patient with steno-occlusive cerebrovascular diseases	Germany	N.A	N.A	Automatic Vascular Segmentation	U-Net Architecture	Provides high-performance vascular segmentation solutions for cerebrovascular disease patients	(48)
	19 patients with coronary artery diseases	Iran	Average age: 58.4 years	11 men and 8 women	Vascular Segmentation Optimization	Texture Characterization Features Combined with U-Net	Improves coronary artery disease vascular segmentation	(49)
	80 patients with intracranial atherosclerosis disease (*n* = 74, *n* = 3, *n* = 3 for training, validation, and testing)	USA	N.A	N.A	Precise Vessel Wall Segmentation	a 2.5D UNet model implemented with a ResNet backbone	Achieves precise intracranial vessel wall segmentation by integrating class inclusion information	(50)
Lesion Detection and Qualitative Analysis	392 patients with CVT (*n* = 294) or without CVT (N = 98)	China	With CVT: 37 ± 14 years; without CVT: 42 ± 15 years	With CVT: 151 women, 153 men; without CVT: 65 women, 33 men	Cerebral Venous Thrombosis Detection	A multisequence multitask DL algorithm	Detects cerebral venous thrombosis using conventional brain MRI	(51)
	421 cases (*n* = 129 with MCA strokes, *n* = 77 with posterior circulation strokes, *n* = 65 watershed strokes, *n* = 150 normal controls)	Turkey	Total: 66.95 (32–95) years [MCA strokes: 67.66 (32–94) years; posterior circulation strokes:69.7 (42–89) years; watershed strokes: 68.35 (37–92) years; normal: 64.33 (32–95) years]	total: 221 male, 200 female [MCA strokes: 68 male, 61 female; posterior circulation strokes:43 male, 34 female; watershed 40 male, 25 female; normal: 70 male, 80 female]	Stroke Region Classification	Deep Learning Model	Performs stroke detection and vascular region classification based on diffusion-weighted MRI	(52)
	1,136 patients with AIS (n = 986 in the training and internal validation cohorts, *n* = 150 in the external validation cohort)	China	Training and internal validation cohorts: 55 (47–65) years; external validation cohort: 63 (53–75) years	Training and internal validation cohorts: 664 males, 322 females; external validation cohort: 100 males, 50 females	Acute Ischemic Stroke Identification	50-layer Residual Network (ResNet 50)	Automatically detects acute ischemic stroke using non-contrast CT	(53)
Quantitative Parameter Extraction	453 patients with AIS (*n* = 345 in the internal cohort, *n* = 108 in the external cohort)	China	Internal cohort: 67 ± 2 (59–73) years; external cohort:61 ± 4 (50–73)	Internal cohort: 188 males, 157 females; external cohort: 71 males, 37 females	Ischemic Core Identification	End-to-end 3D U-net architecture	Identifies acute ischemic core and perfusion deficit regions from non-contrast CT and CTA	(54)
	103 patients with ischemic stroke (dataset from ISLES challenge 2018)	N.A	N.A	N.A	Lesion Segmentation	SLNet: end-to-end U-Net architecture	Automates ischemic stroke lesion segmentation via CT perfusion image synthesis	(56)
	Datasets from ISBI 2015 (Multiple sclerosis lesion, *n* = 5 patients), ISLES 2015 (ischemic stroke, *n* = 28 labeled MRI scans), and BRATS 2018 (brain tumor, 285 training MRI scans)	N.A	N.A	N.A	Multiple sclerosis lesion, ischemic stroke lesion, and brain tumor segmentation	Semi-Supervised Learning	Performs brain lesion segmentation using multi-scale mean teacher combined with adversarial networks	(55)
	datasets from 2015 ISLES challenge: sub-acute ischemic stroke segmentation (SISS) sub-task (28 training and 36 testing cases); acute stroke penumbra estimation sub-task (SPES) (30 training and 20 testing cases)	N.A	N.A	N.A	Multimodal MRI Segmentation	an asymmetrical residual CNN based on the U-Net architecture	Segments acute and subacute stroke lesions from multimodal MRI	(127)
Risk Prediction and Prognosis Assessment	307 patients with VCI: model construction dataset, *n* = 154 in VCI group, *n* = 153 in CVD patients with normal cognition (NC); external validation dataset (*n* = 74 in VCI group, *n* = 83 in NC group)	China	Model construction dataset: VCI group 64 [58–68] years, NC group 59 [55–63.5] years external validation dataset VCI group 68.5 [66–71] years, NC group 63 [60–69] years	Model construction dataset: VCI group 58 females, NC group 65 females; external validation dataset: VCI group 30 females, NC group 35 females	Vascular Cognitive Impairment Diagnosis	Multimodal CNN Framework that combined the vision transformer and extreme gradient boosting algorithms	Integrates multi-source information for vascular cognitive impairment diagnosis	(57)
	multilpe imaging datasets: MIDAS (healthy, *n* = 109); OASIS3 (healthy, *n* = 66); Stroke (*n* = 100); CTA (healthy, *n* = 10)	USA, Germany, Switzerland	N. A	MIDAS (healthy, female *n* = 57); OASIS3 (healthy, female *n* = 29); Stroke (female *n* = 54); CTA (female *n* = 4)	Stroke Classification	U-Net Architecture	Conducts end-to-end stroke classification using cerebrovascular morphological features	(58)
	324 patients with anterior circulation large vessel occlusion treated with mechanical thrombectomy: derivation cohort (*N* = 250); validation cohort (n = 74)	Japan	Derivation Cohort (74.4 ± 11.5 years); Validation Cohort (75.7 ± 11.7 years)	Derivation Cohort (females *n* = 104); Validation Cohort (females *n* = 35)	Clinical Outcome Prediction	Multimodal CNN Framework	Predicts clinical outcomes in large vessel occlusion patients using deep learning-derived features	(61)
	182 patients with acute ischemic stroke: *n* = 32 with minimal reperfusion, *n* = 41 with partial reperfusion, *n* = 67 with major reperfusion, and *n* = 42 with unknown reperfusion	USA	65 ± 15 years	male *n* = 85, female *n* = 97	Final Infarct Core Prediction	U-Net Architecture	Predicts final ischemic stroke lesion from initial MRI	(60)
	Datasets from 2015 ISLES challenge: sub-acute ischemic stroke segmentation (SISS) sub-task (28 training and 36 testing cases); acute stroke penumbra estimation sub-task (SPES) (30 training and 20 testing cases)	N.A	N.A	N.A	Treatment Outcome Prediction	3D U-Net architecture	Predicts outcomes of endovascular treatment in acute ischemic stroke patients	(126)

In the phase of image preprocessing, DL methods exhibit remarkable efficacy in enhancing the quality of medical images. Eun et al. enrolled 14 healthy volunteers (including 7 men and 7 women) from South Korea who had undergone compressed sensing MRI. They used the basic structure of U-Net as a denoising network. These DL-based image quality enhancement approaches can be applied to compressed sensing MRI (CS-MRI). Specifically, self-supervised and unsupervised DL models have been validated to effectively improve the quality of vessel wall images while mitigating the interference of artifacts (45). Moreover, DL reconstruction techniques enable the generation of high-quality 3D T1-weighted sampling perfection with application-optimized contrasts via different flip angle evolution (3D T1-SPACE) sequence vessel wall images. On the basis of tests on images from 10 healthy volunteers and 5 consecutive patients, these techniques not only maintained or even increased image quality but also significantly reduced scan time. This method might overcome the long-standing challenge of balancing imaging efficiency and quality (46). Ota et al. enrolled 12 healthy subjects (6 males and 4 females) and 2 patients (1 male and 1 female) with coronary artery disease in Japan. By targeting the motion artifact issue commonly encountered in MRI, DL reconstruction algorithms—when combined with the design of specific k-space trajectories—have been shown to substantially enhance the motion robustness of coronary magnetic resonance angiography (CMRA) (47).

Automated vessel segmentation and 3D reconstruction are key tasks in DL applications. Livne and colleagues enrolled 66 patients with steno-occlusive cerebrovascular diseases in Germany. The U-Net model has proven effective in automatic vascular segmentation of cerebral vessels in patients, thus offering a dependable tool for the diagnosis of diseases (48). In a cohort of 19 patients (11 men and 8 women) with coronary artery diseases in Iran, the combination of texture characterization features and the U-Net model improved vascular segmentation in coronary artery disease, indicating its potential in automated vascular disease assessment (49). Zhou et al. enrolled 80 patients with intracranial atherosclerosis disease in the USA and developed a 2.5D UNet model implemented with a ResNet backbone. This DL model significantly enhances segmentation accuracy by effectively addressing the class imbalance and boundary ambiguity issues inherent in intracranial vessel wall imaging (50). These segmentation results provide essential data support for subsequent 3D vessel wall reconstruction and visualization, facilitating intuitive anatomical understanding and quantitative assessment of vascular abnormalities.

DL algorithms have demonstrated substantial improvements in lesion detection and qualitative analysis. For example, Yang et al. enrolled 392 patients with cerebral venous thrombosis (CVT) (*n* = 294) or without CVT (*N* = 98) in China. The authors developed a multisequence multitask DL algorithm for detecting CVT on the basis of routine brain MRI data. This DL model achieved an AUC of 0.96, a sensitivity of 96%, and a specificity of 88% at the per-patient diagnosis level. Additionally, a sensitivity of 88% and a specificity of 80% were achieved by this model at the per-segment diagnostic level. Compared with radiologists, this DL algorithm has greater sensitivity at both the per-patient and per-segment diagnostic levels (51). In a cohort of 421 cases (n = 129 with MCA strokes, *n* = 77 with posterior circulation strokes, n = 65 watershed strokes, n = 150 normal controls) in Turkey, Cetinoglu and colleagues developed a DL model based on the MobileNetV2 and EfficientNet-B0 CNN architectures. This model is capable of identifying lesions on DWI and classifying vascular territories, thereby facilitating the rapid localization and characterization of ischemic lesions (52). Additionally, Lu et al. enrolled 1,136 patients with acute ischemic stroke (AIS) in China (*n* = 986 in the training and internal validation cohorts, *n* = 150 in the external validation cohort). They developed a two-stage DL model to overcome the challenge of identifying occult acute ischemic stroke via noncontrast computed tomography (NCCT), a modality where subtle ischemic changes are often indistinguishable to the human eye. With the assistance of this model, the diagnostic accuracy for occult AIS was significantly improved (53).

In terms of quantitative parameter extraction, multiscale 3D CNN models have demonstrated the ability to identify acute ischemic cores and ischemic regions from NCCT and CTA images. For example, Wang et al. enrolled a cohort of 453 patients with AIS in China. They adopted an end-to-end 3D U-net architecture to identify the ischemic core. This model has high potential for objective quantification of the extent of ischemic lesions—a critical basis for guiding clinical intervention strategies (54). For brain lesion segmentation, Chen and colleagues developed a semisupervised approach that integrates a multiscale mean teacher, an adversarial network, and shape-aware embedding. On the basis of public brain lesion datasets from ISBI 2015 (multiple sclerosis lesions, n = 5 patients), ISLES 2015 (ischemic stroke, n = 28 labeled MRI scans), and BRATS 2018 (brain tumor, 285 training MRI scans), this method achieves precise segmentation even with limited annotated data by leveraging unlabeled samples and incorporating shape prior knowledge to constrain segmentation boundaries (55). Additionally, Wang et al. developed a deep neural network based on an attention mechanism. On the basis of datasets from 103 patients with ischemic stroke (dataset from ISLES challenge 2018), this model automatically segments ischemic stroke lesions in CTP images through image synthesis technology—addressing the challenge of ambiguous lesion boundaries in perfusion imaging and improving the accuracy of quantitative lesion assessment (56).

In the segment of risk prediction and prognostic evaluation, DL models have been developed to construct personalized prediction tools by fusing imaging features with clinical data. Fan et al. enrolled a total of 307 patients with CVD (*n* = 154 in the vascular cognitive impairment (VCI) group, *n* = 153 in the CVD patients with normal cognition (NC) group) in China. They developed a DL model based on the Vision Transformer (ViT) and an ML model utilizing clinical nonimaging data. A comprehensive hybrid model was constructed by integrating the ViT model and clinical nonimaging features. This hybrid model achieved an AUC of 0.965 and an area under the precision–recall curve (AP) of 0.972. In the external validation set (157 subjects, *n* = 74 in the VCI group, *n* = 83 in the NC group), the model demonstrated an AUC and AP of 0.902 and 0.900, respectively. These findings indicate that DL-based frameworks integrating multimodal data (e.g., structural imaging, functional imaging, and clinical demographics) can be applied to the diagnosis of vascular cognitive impairment, enhancing the discriminative ability between vascular cognitive impairment and other cognitive disorders (57, 58).

Moreover, ML methods have achieved end-to-end stroke triage by leveraging cerebrovascular morphological features, streamlining the process of prioritizing patients requiring urgent intervention (58). Wouters et al. enrolled 27 patients from the MR CLEAN (derivation) and 101 patients from the CRISP study (validation) in Europe. They developed a DL model for predicting the final infarct size in AIS patients with large vessel occlusions in the anterior circulation. Compared with computed tomography perfusion imaging processing software, this DL model exhibited improved prediction of the final infarct volume, thus providing a quantitative basis for determining the window of opportunity for reperfusion therapy (59). Yu et al. enrolled 182 patients with AIS (*n* = 32 with minimal reperfusion, *n* = 41 with partial reperfusion, *n* = 67 with major reperfusion, and *n* = 42 with unknown reperfusion) in the USA. They developed a DL model based on the U-Net architecture and demonstrated that this model can predict the final ischemic stroke lesion volume, with a diagnostic performance comparable to that of conventional clinical assessment methods (60). Nishi enrolled 324 patients with anterior circulation large vessel occlusion who were treated with mechanical thrombectomy in Japan. They developed a DL model based on a 3D-U-Net architecture. This model had the highest AUC of 0.81 compared with 0.63 and 0.64 for the Alberta Stroke Program early CT score and ischemic core volume models, respectively. This DL model has been validated to predict clinical outcomes, such as functional independence at 90 days post-treatment, in patients with large vessel occlusion (61). These advancements collectively highlight the value of DL in personalized prognostic evaluation and provide crucial references for evidence-based clinical decision-making.

## 3 Research on HR-VWI in intracranial vascular lesions

### 3.1 Atherosclerosis

Atherosclerosis represents one of the major etiologies of ischemic stroke, and radiological assessment of plaque stability and rupture risk is directly linked to strategies for secondary prevention and therapeutic decision-making. Unlike traditional imaging modalities that only enable the evaluation of luminal stenosis, HR-VWI offers a unique perspective for identifying vulnerable plaques and assessing stroke risk by directly visualizing the pathological features of the vascular wall (Table 2). The HR-VWI is capable of identifying a variety of features associated with plaque vulnerability. A thin or ruptured fibrous cap, coupled with a large lipid core, is widely recognized as a critical hallmark of vulnerable plaques (62, 63). Intraplaque hemorrhage (IPH), which typically manifests as a hyperintense signal on T1-weighted images, is strongly correlated with plaque instability (62). Compared with male patients, women with atherosclerosis often have more calcified, less lipid-rich plaques (64). Sun et al. enrolled 14 patients (13 males and 1 female) with IPH from the USA. They revealed that carotid plaques with IPH demonstrate a significantly accelerated progression rate, suggesting that hemorrhagic events may expedite the atherosclerotic process (65). Liu et al. enrolled 687 recruited patients (62.7 ± 10.1 years; 69.4% males) with carotid plaques from China. Compared with patients without ACI, those with ACI had a significantly greater BMI, younger age, higher prevalence of males, and larger volume of carotid IPH (66). Compared with other ancestries, there is a greater prevalence of intracranial atherosclerotic disease (IAS) among stroke patients of Asian (approximately 50%), Black (approximately 33%), and Hispanic ancestry (approximately 15%) (67). The high prevalence of diabetes mellitus (DM), smoking, hypertension, hypercholesterolemia, and metabolic syndrome among those populations might partly explain the high prevalence of IAS (68, 69). Collectively, these compositional features form a crucial basis for evaluating plaque stability.

**Table 2:** Applications of HR-VWI in intracranial vascular lesion research.

Disease type	Key HR-VWI features	Critical clinical significance	Ref.
Atherosclerosis	Intraplaque hemorrhage (T1WI high signal), thin or ruptured fibrous cap, positive remodeling (outward expansion), negative remodeling (inward contraction), plaque enhancement	Identification of vulnerable plaques and stroke risk stratification; plaque distribution related to perforator infarction; plaque enhancement as a marker of inflammation activity and recurrence risk	(62, 63, 76–78)
Stroke risk prediction and recanalization	Elevated plaque burden and enhancement ratio; culprit plaque enhancement; post-treatment vessel wall morphological changes; residual lumen in occlusion segment and shorter occlusion length	Predict stroke recurrence; select candidates for endovascular recanalization; assess feasibility of recanalization and treatment prognosis	(80–82, 84, 85)
Intracranial aneurysm	Aneurysm wall enhancement correlated with morphological complexity and hemodynamic parameters; anti-inflammatory drug (e.g., aspirin) may reduce enhancement	Assess rupture risk and post-treatment recanalization risk; guide anti-inflammatory therapy	(87–89)
Arterial dissection	Intimal flap, double lumen sign, intramural hematoma; chronic phase vessel wall remodeling and signal evolution	Definitive diagnosis of dissection; distinguish dissecting aneurysm from segmental dilation; evaluate vessel wall changes at different dissection stages	(91, 92, 94)
Central nervous system vasculitis	Smooth, concentric wall thickening with uniform enhancement of small/medium arteries; reduction in enhancement during follow-up under therapy	Differentiate from atherosclerosis; dynamically monitor disease activity and therapeutic response	(95, 97, 98)
Moyamoya Disease (MMD)	Progressive stenosis/occlusion of terminal ICA, vessel wall thickening and thinning of media; smoky collateral networks; wall enhancement correlated with disease stage and infarction events	Assess disease progression and risk of complications; distinguish MMD from atherosclerotic moyamoya syndrome	(101, 102, 128, 129)
Arteriovenous malformation (AVM)	Abnormal high signal within the nidus vessel walls, enhancement of draining veins, venous wall enhancement	Suggest abnormal vessel wall structure and inflammation; assess hemodynamic status and hemorrhage risk	(105–107)

The morphological characteristics and distribution patterns of plaques also hold substantial clinical significance. Vascular remodeling refers to the adaptive changes occurring in blood vessels during the development of atherosclerosis (62, 70). Positive remodeling is characterized by outward expansion of the vascular wall, which may maintain luminal patency. However, this remodeling pattern is associated with increased plaque vulnerability and a greater risk of microembolism. In contrast, negative remodeling involves inward contraction of the vascular wall, which often leads to significant luminal stenosis. The location of plaques on the vascular wall also influences clinical outcomes. HR-VWI analyses have revealed that plaques located at the orifice of perforating arteries may directly occlude these orifices, thereby increasing the risk of infarction in the perforating artery territory (71). A retrospective study enrolled 22 patients (14 males, 58.8 ± 13.0 years) with symptomatic atherosclerosis and 24 asymptomatic patients (18 males, 62.4 ± 12.3 years) from China. This study revealed that, among patients with mild middle cerebral artery stenosis, contrast enhancement and superior distribution of culprit plaques (i.e., plaques located on the ipsilateral side of the stroke) are associated with recent ischemic stroke, indicating that such plaques may still induce prominent clinical symptoms despite mild luminal narrowing (72). These findings underscore the pivotal role of the spatial distribution characteristics of plaques in the mechanisms underlying stroke.

The assessment of plaque activity is crucial for predicting clinical outcomes. The progression of plaque volume and burden is correlated with recurrent ischemic events (73). Plaque enhancement, especially the obvious enhancement observed in contrast-enhanced scans, is generally considered to reflect intraplaque inflammatory activity or neovascularization and is associated with an increased risk of recent ischemic symptoms and stroke recurrence (62). A prospective study by Yang et al. enrolled 58 patients with stroke or transient ischemic attack (mean age: 64 years; 35 males: 60%) from Hong Kong, China. This study revealed that in acute lesions, symptomatic plaques exhibit significantly stronger enhancement than asymptomatic plaques do; moreover, in patients with symptomatic ICAD, a reduction in plaque enhancement after intensive treatment is associated with improved clinical prognosis (74). Fakih et al. enrolled 344 patients with cryptogenic stroke from the USA, 34 of whom received 7 T HR-VWI MRI. The detection and quantification of symptomatic atherosclerotic plaques via 7 T HR-VWI help identify the etiology of stroke. Culprit plaques are characterized by greater contrast enhancement, more severe stenosis, and a concentric morphology (75).

These dynamic features provide potential indicators for monitoring disease progression and treatment response. With technological advancements, quantitative analysis and automatic segmentation methods for vascular walls have been continuously developed. Neural network-enhanced three-dimensional turbo spin–echo sequences have improved the quality and efficiency of intracranial vascular wall imaging (76). Wu and colleagues developed a DL model (named the proposed DeepMAD network) based on a modified U-shaped convolutional network (U-Net). This model was trained on the CAREII dataset, which includes 1,057 carotid MR images collected from 13 hospitals and medical centers in China. The AIM-HIGH trial dataset, which includes 425 carotid MRI scans collected at 21 clinical sites in China, was used as the test set. The DeepMAD model performs automated segmentation of the carotid artery wall and provides diagnostic support for atherosclerosis (77). Ma et al. enrolled 40 patients with atherosclerotic plaques in the intracranial or carotid artery in China. AI-constrained compressed sensing accelerates the acquisition of 3D isotropic T1-VISTA sequences, demonstrating the potential to substantially reduce scan times without compromising image quality (78). Furthermore, Wang et al. enrolled 115 patients (120 carotid arteries) from China. They developed a multichannel shape-aware 3D nnU-net model for segmenting the vessel wall of the carotid artery. This model allows simultaneous analysis of multisequence MR images, enabling integrated vascular wall segmentation and plaque component characterization (79). Together, these developments offer robust support for the precise quantitative assessment of atherosclerosis.

### 3.2 Evaluation of revascularization treatment strategies

HR-VWI offers both qualitative and quantitative insights into intracranial vascular structures, making it increasingly valuable for assessing stroke risk, evaluating treatment prognosis, and guiding patient selection and strategy development for vascular recanalization. When predicting the likelihood of stroke recurrence, specific vessel wall characteristics observed on HR-VWI have demonstrated a strong correlation with subsequent ischemic events. For example, Lv et al. enrolled 132 patients with symptomatic ICAD (mean age: 59.83 ± 11.06 years; 46.2% female and 53.8% male) in China. Quantitative 3D HR-MRI analyses indicate that increased plaque burden and increased plaque enhancement ratios serve as independent predictors of long-term stroke recurrence in symptomatic ICAD patients (80). A prospective study enrolled 169 patients (70 (65, 73) years, 62.9% male) with large atherosclerotic ischemic stroke from China. This study confirmed that the total number of intracranial and extracranial carotid plaques, as well as the coexistence of extracranial carotid plaques and significantly enhanced intracranial plaques, are independent risk factors for ischemic stroke recurrence (81). Such plaque enhancement is considered to reflect the active inflammatory process or neovascularization of the lesion. Furthermore, HR-VWI can detect culprit plaques in cases where luminal stenosis is insignificant. A study based on 3D HR-VWI demonstrated that plaque volume and the total plaque enhancement ratio are significantly associated with plaques implicated in stroke. The characteristics of these culprit plaques on 3D HR-VWI have potential for predicting risk in patients whose ischemic stroke stems from arteries without major stenosis (82). These findings indicate that HR-VWI has substantial potential in identifying high-risk individuals and directing targeted, intensive secondary prevention.

HR-VWI findings are also correlated with recanalization outcomes and long-term functional recovery. In acute stroke patients, the vascular wall changes observed following recanalization therapy have important clinical implications. One study enrolled 29 patients with stroke from Korea and explored vascular wall changes following recanalization therapy for acute stroke and reported that HR-VWI can visualize concentric enhancement of the vascular wall posttreatment, which is associated with hemorrhagic transformation (83). Chao et al. enrolled 75 patients with ipsilateral recurrent ischemic stroke (53 men, mean age 57.51 ± 9.71 years) in China and investigated the feasibility of HR-VWI-guided endovascular recanalization therapy for nonacute intracranial arterial occlusion (ICAO). The study revealed that preprocedural HR-VWI evaluation of luminal and vessel wall alterations could predict the likelihood of successful endovascular recanalization, thereby supporting more precise interventional planning (84). These results underscore the clinical value of HR-VWI in selecting appropriate candidates and optimizing revascularization protocols. In symptomatic internal carotid artery occlusion (ICAO) patients, endovascular recanalization has been shown to improve outcomes. HR-VWI enables qualitative and quantitative characterization of the occluded segment—including residual lumen patency and occlusion length—which aids in assessing the technical feasibility of recanalization (85). Comparative analyses further indicate that HR-VWI supplements digital subtraction angiography (DSA) by improving patient selection for endovascular treatment of carotid tandem occlusions (86). These findings support the use of HR-VWI as a key tool for preoperative evaluation and individualized revascularization strategy design.

### 3.3 Intracranial aneurysms and arterial dissections

IAs are localized outpouchings of the cerebral vasculature that pose a significant risk of rupture and life-threatening hemorrhage. In IA evaluation, aneurysm wall enhancement (AWE) observed on HR-VWI is correlated with underlying inflammatory activity and hemodynamic stress. Huang et al. studied 100 patients (112 unruptured aneurysms) in China and reported that the intensity of AWE in unruptured IAs corresponds to more complex aneurysm morphology and irregular hemodynamic patterns (87), potentially reflecting greater wall inflammation and structural instability. In terms of clinical relevance, patients on long-term daily aspirin (≥6 months) demonstrate less AWE, implying that anti-inflammatory medication may modify aneurysm wall biology (88). HR-VWI also holds significant value for identifying aneurysms after treatment. The occurrence of AWE in aneurysms after coil embolization is associated with an increased risk of aneurysm recanalization (89), whereas after treatment with flow diverters, HR-VWI can be used to evaluate the occlusion status of the aneurysm effectively and the patency of the parent artery (90).

Arterial dissection refers to a tear of the arterial intima, where blood enters the arterial wall to form a false lumen, which may lead to vascular stenosis or occlusion. HR-VWI can clearly visualize typical dissecting features, including intimal flaps, double-lumen signs, and intramural hematomas. In the assessment of spontaneous unruptured chronic ICAD, HR-VWI is capable of identifying distinctive morphological characteristics, thereby providing important evidence for diagnosis (91). For vertebrobasilar dissecting aneurysms (VBDAs), which are clinically difficult to diagnose, 3D HR-VWI has practical differential diagnostic value and can accurately distinguish dissecting aneurysms from segmental dilations (92). HR-VWI features are closely associated with treatment decision-making and prognostic assessment. In a prospective study by Hu et al., the preoperative vascular wall imaging patterns of patients with UIAs were found to be correlated with surgical outcomes, and specific enhancement patterns may indicate different treatment responses (93). A retrospective study on cervicocranial arterial dissection (CCAD) demonstrated that HR-VWI can be used to evaluate wall changes at different stages, including the signal characteristics of hematomas and the process of vascular wall remodeling (94). Collectively, these findings establish the critical role of HR-VWI in the individualized diagnosis and treatment of IAs and arterial dissections.

### 3.4 Central nervous system vasculitis

Central nervous system vasculitis (CNSV) is a group of immune-inflammatory diseases that primarily involve the small- and medium-sized blood vessels of the cerebral parenchyma, spinal cord, and leptomeninges. It presents with diverse and nonspecific clinical manifestations; traditional imaging methods rely mainly on indirect signs such as luminal stenosis for diagnosis, making differential diagnosis challenging. Unlike ICAD, which typically manifests as eccentric and irregular vascular wall thickening, the typical HR-VWI feature of CNSV is concentric, smooth vascular wall thickening involving small- and medium-sized arteries, accompanied by uniform enhancement. A study by Cao et al. revealed that HR-VWI is more sensitive than MRA in detecting CNSV, especially in pediatric patients (95). In pediatric CNSV, lesions mainly involve medium-sized vessels, presenting as grade 1 and grade 2 stenosis (grade 4 stenosis is relatively rare), and the imaging features usually show concentric and moderate enhancement—characteristics that are specific to the diagnosis of CNSV (95). In addition, HR-VWI can clearly visualize strong concentric vascular wall enhancement (VWE), which is particularly prominent in large- and medium-sized vessel vasculopathy (LMVV) and small vessel vasculopathy (SVV) (96). Patients with LMVV are more likely to present with strong concentric VWE, whereas patients with SVV more commonly have contrast-enhanced lesions in the meninges or cerebral parenchyma (96). Furthermore, HR-VWI has revealed that changes in vascular wall inflammation are closely associated with cerebral parenchymal lesions and clinical symptoms, which further supports its value in disease assessment (95).

HR-VWI not only facilitates diagnosis but also effectively monitors the therapeutic response of CNSV. A study by Patzig et al. revealed that serial follow-up imaging after treatment revealed that the degree of vascular wall enhancement gradually diminished or disappeared as clinical symptoms improved, whereas the resolution of vascular wall thickening was relatively slow (97). In a study involving 9 CNSV patients who underwent serial HR-VWI examinations, the reduction in VWE during follow-up was associated with a favorable therapeutic response. At baseline, 25.5% of the vascular segments in these patients exhibited strong concentric VWE; however, this proportion decreased significantly to 6.5% during the follow-up period, indicating effective treatment (98). Additionally, in cases of disease recurrence, VWE scores worsened during follow-up; however, after intensive immunosuppressive therapy, VWE scores improved significantly. These findings demonstrate that the HR-VWI can dynamically reflect disease activity, providing a basis for adjusting treatment regimens.

### 3.5 Atherosclerosis and arteriovenous malformation

Moyamoya disease is a cerebrovascular disorder characterized by progressive stenosis/occlusion of the terminal segment of the internal carotid artery and its branches, accompanied by the formation of basal collateral vessels (i.e., “moyamoya vessels”). MMD is more prevalent in East Asian countries, particularly Japan and Korea (99). The diagnosis of MMD relies primarily on luminal changes and moyamoya-like collateral networks visualized via angiography (100). However, these methods are unable to reveal the specific structures and pathological activities of pathological vessel walls. HR-VWI, as reviewed by Yang et al., can clearly reveal features such as vascular stenosis, intimal thickening, and medial thinning in MMD patients (101). Ouyang et al. enrolled 47 patients with MMD from China and retrospectively analyzed the relationships between intravascular enhancement sign (IVES) in HR-VWI and cerebral perfusion indicators. HR-VWI can visualize the degree of vascular wall enhancement in MMD patients, which is closely associated with disease stage (e.g., Suzuki staging); the higher the degree of enhancement is, the faster the disease progresses (102). Similarly, Lu et al. enrolled 170 MMD patients with HR-VWI data from China. They reported that vessel wall enhancement is a prominent feature in MMD patients and is positively associated with rapid progression of arterial stenosis and increased risk for stroke (103). Kim and colleagues enrolled 66 patients with MMD from Korea. Serial HR-MRI indicated that the degree of stenosis increased with negative remodeling (outer diameter shrinkage). Most patients (*n* = 61) showed vascular enhancement. However, follow-up HR-MRI revealed that only 6 patients experienced progression of enhancement, and 1 patient experienced new vascular enhancement (68). In another study enrolling 48 patients (76 hemispheres) from Korea, HR-VWI revealed significant differences in vascular morphology between ruptured and unruptured choroidal collaterals in adult MMD patients. A larger aortic left atrium (>1.285 mm^2^) and simple aortic angiography are independently associated with aortic hemisphere rupture (104). The above studies indicate that the features of the vessel walls of MMD patients may vary, even if they are from the same geographical region.

AVM is composed of abnormal arteriovenous shunts and niduses and is often accompanied by complex changes in perfusion and venous drainage. The application of HR-VWI in AVM assessment is relatively preliminary; however, existing studies have shown that HR-VWI can detect enhanced signals in vessel walls within or adjacent to the AVM nidus—even in AVM patients without a history of hemorrhage (105). In a pilot study, Eisenmenger et al. performed HR-VWI on 9 patients with unruptured AVMs and reported that 8 patients presented with abnormally hyperintense signals in the vessel walls of the AVM nidus. Among these 8 patients, 4 had enhancement in more than 50% of the vessel wall area, and 3 had adjacent vessel wall enhancement (105). This observation suggests that structural or inflammatory changes may already exist in AVM vessel walls even in the absence of a clinical history of rupture. A feasibility investigation examined the clinical utility of combining HR-VWI with quantitative blood flow assessment in AVM. The results demonstrated that hemorrhagic AVMs and those with elevated venous volume flow were more frequently associated with enhancing draining veins (106), suggesting that VWI and quantitative flow techniques can detect signal enhancement within AVM venous walls. Further quantitative analysis of AVM draining veins indicated that hemodynamic forces—particularly low wall shear stress—may contribute to inflammatory changes and venous stenosis (107). Collectively, these observations position HR-VWI as a promising technique for characterizing AVM angioarchitecture and hemodynamic status, offering valuable insights to inform therapeutic decision-making.

## 4 Clinical integration and translation of the DL and HR-VWI

### 4.1 Intelligent diagnosis and lesion screening

The integration of DL with HR-VWI is transforming the diagnostic approach to intracranial vascular disease. Embedding DL modules into postprocessing workflows enables rapid identification and characterization of intracranial aneurysms, atherosclerotic plaques, culprit lesions, and vascular territories. This not only reduces the manual workload and improves efficiency but also provides auxiliary judgment in high-throughput screening (Table 3). In terms of automatic IA detection and vascular morphological analysis, DL-based algorithms can directly process 3D MRA data to efficiently complete cerebrovascular segmentation and IA identification. Joo et al. constructed a 3D ResNet architecture algorithm for MRAs to automatically detect IAs. They identified 551 aneurysms (mean diameter, 4.17 ± 2.49 mm) in the training set, 147 aneurysms (mean diameter, 3.98 ± 2.11 mm) in the internal test set, and 63 aneurysms (mean diameter, 3.23 ± 1.69 mm) in the external test set. In the internal test set, the sensitivity, positive predictive value, and specificity were 87.1, 92.8, and 92.0%, respectively; in the external test set, this CNN model achieved a sensitivity of 85.7%, positive predictive value of 91.5%, and specificity of 98.0%. Notably, this model achieved a sensitivity of 84.3% (59/70) in the internal test set and 73.5% (25/34) in the external test set for IAs less than 3 mm. Generally, this model exhibited high diagnostic performance for IAs (108). Another study conducted by Ryu and colleagues included 675 participants, including 189 aneurysm-positive patients and 486 aneurysm-negative patients. They confirmed that the 3D ResNet architecture algorithm achieved patient-level sensitivity (95.2%) and specificity (80.5%). In terms of IA size, the detection sensitivities were 72.3% for lesions <3 mm, 91.8% for those 3–5 mm, and 94.3% for those >5 mm. The AUC was 0.949 (109). Those two studies in Korea also confirmed that the 3D ResNet model has a good ability to detect small aneurysms (less than 3 mm).

**Table 3:** Clinical application examples of DL + HR-VWI integration.

Project	People/subjects	Conutry	Age	Gender	Specific Task	DL Models	Diagnostic performance	Ref.
Intelligent Diagnosis and Lesion Screening	551 aneurysms (mean diameter, 4.17 ± 2.49 mm) in the training set, 147 aneurysms (mean diameter, 3.98 ± 2.11 mm) in the internal test set, 63 aneurysms (mean diameter, 3.23 ± 1.69 mm) in the external test set	Korea	N.A	N.A	Automated detection and localization of intracranial aneurysm	3D ResNet architecture	the internal test set: sensitivity (87.1%), the positive predictive value (92.8%), and the specificity (92.0%); the external test set, sensitivity (85.7%), the positive predictive value (91.5%), and the specificity (98.0%)	(108)
	675 participants [189 aneurysm-positive (221 unruptured intracranial aneurysms)] and 486 aneurysm-negative	Korea	Overall: 59.6 ± 11.3 years. Without IA: 58.2 ± 10.7 years; With IAs:62.1 ± 11.0 years	351 females and 324 males. Without IA: 284 males (58.4%); With IAs: 40 males (21.2%)	Detecting unruptured intracranial aneurysms	3D ResNet architecture	Patient-level sensitivity (95.2%) and specificity (80.5%); lesion-level sensitivity (89.6%) and a false-positive rate of 0.19 per patient. Sensitivity by aneurysm sizes: 72.3% for lesions <3 mm, 91.8% for 3–5 mm, and 94.3% for >5 mm. Performance ROC: 0.949.	(109)
	1806 plaques from 726 patients with symptomatic intracranial atherosclerotic stenosis; training set (998 plaques), validation set (396 plaques), test set (412 plaques)	China	58.0 ± 7.0 years, age range, 32–92 years	492 males and 234 females	Diagnosing vulnerable intracranial atherosclerotic plaques	ResNet50 and Vision Transformer (ViT) architectures	the training set: AUC (0.877 for ResNet50, 0.916 for ViT), sensitivity (78.2% for ResNet50, 83.8% for ViT), specificity (81.2% for ResNet50, 85.0% for ViT); the validation set, AUC (0.879 for ResNet50, 0.927 for ViT), sensitivity (75.2% for ResNet50, 84.0% for ViT), specificity (84.2% for ResNet50, 88.4% for ViT); the test set, AUC (0.845 for ResNet50, 0.913 for ViT), sensitivity (73.8% for ResNet50, 85.1% for ViT), specificity (79.3% for ResNet50, 82.5% for ViT)	(110)
	660 participants (200 MMD, 200 ASD and 200 NC) and 60 from another institution. The training set (*n* = 450), the validation set (*n* = 90), internal testing set (*n* = 60), and external testing set (n = 60)	China	55.2 ± 12.1 years	357 females, 303 males	Vascular segmentation and 3D reconstruction	DenseNet121, ResNet50, SENet154, SEResNet50, and SEResNext50	The mACCs: 0.911 for DenseNet121, 0.887 for Resnet50, 0.867 for SENet154, 0.922 for SEResNet50, and 0.833 for SEResNext50. DenseNet-121 exhibited superior discrimination capabilities: AUC = 0.977 in the test sets and 0.870 in the external validation sets;	(111)
	129 consecutive patients with atherosclerotic plaques	China	Age range 46–78 years, mean age 58.6 ± 18.9 years	N.A	Segmentation of arterial vessel wall and plaque	CNN-based VWISegNet	The Dice similarity coefficient (DSC) of 93.8% for lumen contours and 86.0% for outer wall contours; average surface distance (ASD) values were less than 0.198 mm. coefficient values between the automatic method and manual method were greater than 0.780	(112)		1806 plaques from 726 patients with symptomatic intracranial atherosclerotic stenosis; training set (*n* = 400), validation set (*n* = 158), test set (*n* = 168)	China	58.0 (51.0, 66.0) years	492 males and 234 females	Responsible plaque determination	Habitat Radiomics + Vision Transformer (Habitat + ViT)	In the validation set, the model achieved an AUC of 0.949, a sensitivity of 0.879, a specificity of 0.905, and an accuracy of 0.897. In the test set, the AUC increased to 0.960, with specificity rising to 0.963 and an accuracy of 0.885; plaque heterogeneity features predict stroke events	(113)
1981 patients undergoing 3D MR-VWI examinations: training set (*n* = 524), validating set (*n* = 104), testing set (*n* = 120), clinical evaluation set (*n* = 134), and application data set (*n* = 1,099)	China	61 ± 12 years	686 females and 1,295 males	Rapid vessel segmentation and reconstruction	Multi-sequence integrated DL platform	achieve 92.9% qualified rate of manual delineation; reduce processing time by over 90% (10–12 min per case); improve inter−/intra-reader agreement. Real-world deployment (n = 1,099 patients) demonstrated rapid clinical adoption, with utilization rates increasing from 10.8 to 100.0% within 12 months.	(114)		
539 patients with cerebrovascular disease: training set (*n* = 432), testing set (*n* = 55), and evaluation dataset (*n* = 52)	China	11–94 years; mean age: 56 ± 14 years	Female: 164; male: 374	Vessel centerline extraction	3D V-Net network	The average detection accuracy was 88.99%, accuracy in the internal carotid artery and middle cerebral artery was 95.4%, the accuracy at the sharp bend of the carotid siphon section reached 97%, the accuracy in detecting the points in the internal carotid artery and middle cerebral artery was 95.4%, the ACD for the right carotid artery was reduced to 0.484 ± 0.321 mm, time required to detect the 32 key points was reduced from 319.843 ± 6.434 to 2.046 ± 0.315 s	(115)		
Prognosis Prediction	235 patients (132 symptomatic plaques and 103 asymptomatic plaques): training set (*n* = 156) and a testing set (*n* = 79).	China	57 (49.5, 64) years	78 females, 157 males	Symptom-related plaque identification	Radiomics model in combination with DL models (DenseNet 121, DenseNet 201, GoogleNet, ResNet 18, and ResNet 34)	DL models achieved an AUC of 0.947 (the training set) and 0.956 (testing set), accuracy of 87.8% (the training set) and 89.9% (testing set), sensitivity of 88.9% (the training set) and 95.2% (testing set), and specificity of 86.4% (the training set) and 83.8% (testing set). The combination of DL models with Traditional model achieved better performance in identifying plaques	(116)
	28 subjects underwent at least three VWIs over a span of at least 5 years	USA	71.3 ± 9.7 years	20 males and 8 females	Vessel wall and intraplaque hemorrhage (IPH) segmentation	3D SonoNet	DL-based segmentation pipelines were utilized to identify IPH, quantify IPH volume, and measure their effects on carotid plaque burden during long-term follow-up	(117)	
363 patients with symptomatic intracranial atherosclerotic stenosis (sICAS): training set (*n* = 254), test set (*n* = 109)	China	Training set (*n* = 254): No recurrence [*n* = 204, 58.0 (51.0, 66.0) years], recurrence [*n* = 50, 58.0 (50.0, 66.0) years]; Test set (*n* = 109): No recurrence [*n* = 84, 58.0 (51.0, 67.0) years], recurrence [*n* = 25, 58.0 (47.0, 63.0) years]	Training set (*n* = 254): No recurrence (*n* = 204, 63 males, 141 females), recurrence (*n* = 50, 19 males, 31 females); Test set (*n* = 109): No recurrence (*n* = 84, 28 males, 56 females), recurrence (*n* = 25, 7 males, 18 females)	Capture image information from culprit plaques in HR-VWI; predict stroke recurrence in sICAS patients	ResNet50, DenseNet169, and Trans-CNN	Trans-CNN model achieved an AUC of 0.951, accuracy of 0.880, sensitivity of 0.900, and specificity of 0.882 in training set, and achieved an AUC of 0.912, accuracy of 0.858, sensitivity of 0.880, and specificity of 0.810 in the test set. Trans-CNN had better AUC improvement than other two CNN models.	(118)		
99 cases containing 120 aneurysms (91 unruptured, 29 ruptured)	China	Ruptured (*n* = 29): 54.20 ± 13.10 years; unruptured (*n* = 91): 55.75 ± 10.37 years	Ruptured (*n* = 29): 12 females, 17 males; unruptured (*n* = 91): 60 females and 31 males	Aneurysm rupture risk prediction	Backbone network of a 3D ResNet18 followed by a fully connected layer	By integrating with clinical information, the CNN model achieved an AUC of 0.853, Sensitivity of 0.893, Specificity of 0.838, PPV of 0.692, NPV of 0.967, accuracy of 0.853 in aneurysm rupture prediction; enhances clinician prediction performance from AUC = 0.877 to 0.945	(119)		
640 patients with acute ischemic stroke: training set (*n* = 448), validation set (*n* = 96), and internal testing set (*n* = 96); 280 patients from LUH generalization cohort	USA	Training and Validation Cohort (*n* = 544): 69 (57, 78) years	Training and Validation Cohort (*n* = 544): 271 males and 273 females	functional prognosis prediction	a 3D ResNet	Predicts ordinal mRS score and unfavorable outcome in internal/external cohorts	(120)	
363 patients with sICAS. 79 cases of stroke recurrence (21.76%) and 284 cases without recurrence (78.24%). The cohort was divided into Training set (n = 232), validation set (n = 58), and Test set (n = 73).	China	57.57 ± 11.55 years	246 females, 117 males	Predicting stroke recurrence	Multi-View Deep Survival Combined Model	The combined model of automated multi-view deep feature learning and DeepSurv-based survival analysis achieved a C-index of 0.872 in the internal validation set and 0.803 in the external test set; additionally, the AUC of predictive accuracy for 1, 2, and 3-year recurrence was reached to 0.841, 0.870, and 0.802, respectively	(121)
Assist decision-making	23 patients with AVM: training set (*n* = 17, 6,362 image slices) and validation set (*n* = 6, 1,224 slices)	USA	N.A	N.A	AVM radiosurgery planning	2D U-Net-based multiparametric MRI segmentation	Accurate delineation of nidus (artery/vein/brain parenchyma Dice = 0.86/0.91/0.98)	(122)
	236 patients with 266 intracranial aneurysms (IAs): primary cohort (189 patients, 213 IAs, including 149 stable IAs and 64 unstable IAs), Validation cohort (48 patients, 53 IAs, including 35 stable IAs and 18 unstable IAs)	China	Primary cohort (stable IAs: 59.82 ± 9.12 years, unstable IAs: 56.75 ± 12.50 years), Validation cohort (stable IAs: 55.14 ± 12.16 years, unstable IAs: 54.72 ± 7.68 years)	Primary cohort (stable IAs: 101 females, unstable IAs: 43 females), Validation cohort (stable IAs: 25 females, unstable IAs: 10 females)	Aneurysm instability prediction for treatment strategy	MicroAB-Net (4D-Flow MRI + HR-MRI)	4D-Flow-LR achieved an AUC of 0.809, and MicroAB-Net achieved an AUC of 0.807. The Hybrid Model, which combines 4D-Flow-LR and MicroAB-Net, achieved the best performance with an AUC value of 0.854	(123)
	193 patients undergoing examination of high-resolution black-blood MR-VWI: Training dataset (n = 146), External dataset (n = 47)	China	60.2 ± 4.3 years (61.7 ± 10.5 years for training set and 59.6 ± 9.9 years for external dataset)	Training dataset (97 males, 49 females), External dataset (30 males, 17 females)	Vessel wall segmentation	Res U-net	On the internal test set from Center A, the model achieved DSC of 0.936 for the outer wall (DSCA), 0.942 for the lumen (DSCL), and 0.845 for the vessel wall region (DSCW) On the external test set, the model achieved DSCs of 0.928 (outer wall area), 0.936 (lumen area), and 0.844 (vessel wall region)	(124)

When DL models are extended to integrate HR-VWI, they can also classify vulnerable intracranial atherosclerotic plaques simultaneously, thereby improving diagnostic efficiency. For example, Li et al. enrolled 1806 plaques from 726 patients with symptomatic intracranial atherosclerotic stenosis in China. Two DL models, ResNet50 and ViT, exhibited good performance. The ResNet50 model achieved an AUC of 0.879, a sensitivity of 0.752, a specificity of 0.842, and an accuracy of 0.804 in the validation set. In contrast, the ViT model achieved an AUC of 0.927, sensitivity of 0.840, specificity of 0.884, and accuracy of 0.865, suggesting better performance than ResNet50 (110). Lu et al. developed a DenseNet-121 model based on MRA images from patients with MMD (*n* = 200), atherosclerotic disease (ASD, *n* = 200), or normal controls (NCs, *n* = 200) in China. The analysis results revealed that the macroaverage accuracies were 0.911 for DenseNet121, 0.887 for ResNet50, 0.867 for SENet154, 0.922 for SEResNet50, and 0.833 for SEResNext50. Compared with other CNN models, DenseNet-121 achieved an AUC of 0.977 in the internal test sets and 0.880 in the external validation sets, suggesting its superior discrimination capabilities. The authors also performed subgroup analysis stratified by age and sex and verified that the classification performance of the CNN models in MMD, ASD, and NC patients was not significantly different across different age or sex groups. These results indicate that CNN-based DL models improve the accuracy and efficiency of MMD detection and reduce the workload of radiologists (111).

In the fields of culprit plaque identification and vascular territory division, DL models can accurately recognize the culprit plaques causing ischemic stroke by extracting information on plaque morphology, texture, and spatial distribution contained in HR-VWI. Xu et al. obtained MRVWI images acquired from 124 patients with atherosclerotic plaques. They proposed a VWISegNet model for pixel-level classification of arterial walls and plaques. Compared with the traditional U-Net architecture, the VWISegNet model has more residual units, which makes it easier to propagate information between low and high levels, thus alleviating the vanishing gradient problem. VWISegNet achieved better segmentation performance, with a Dice similarity coefficient (DSC) of 93.8% for lumen contours and 86.0% for outer wall contours, and the average surface distance (ASD) values were less than 0.198 mm. A coefficient value over 0.780 was identified when comparing the automatic method and manual method (112). Gao et al. performed a retrospective study enrolling 1806 intracranial plaques from 729 patients (median age: 58.0 (51.0, 66.0) years; 492 males and 234 females) with symptomatic intracranial atherosclerotic stenosis. The included patients were divided into a training set (*n* = 400), a validation set (*n* = 158), and a test set (*n* = 168). These patients were from four center hospitals in China. They combined the Radiomics model with the Vision Transformer (ViT) model and constructed a Habitat + ViT fusion model. In the training set, the Radiomics model, Vit model, and fusion model achieved AUCs of 0.874, 0.916, and 0.953, respectively; in the validation set, the AUCs of those models were 0.839, 0.927, and 0.949; and in the test set, the AUCs of those models were 0.857, 0.913, and 0.960, respectively. This integrated Habitat + ViT model has superior performance in identifying high-risk vulnerable plaques in symptomatic intracranial atherosclerotic stenosis (sICAS) patients (113). Zhang et al. built a DL platform called VWI Assistant for training multicenter 3D MR-VWI datasets (involving 1981 patients) in China. Compared with expert manual delineation, this model achieved a 92.9% qualified rate and reduced the processing time by more than 90% (10–12 min for each case). On the basis of real-world deployment in 1099 cases, this platform exhibited rapid clinical adoption, and the utilization rates increased from 10.8 to 100.0% within 1 year (114). Moreover, Zhang et al. enrolled 539 patients with cerebrovascular disease. By constructing a 3D V-Net network, they reported that this model is able to automatically and accurately detect the internal carotid artery and middle cerebral artery, improving vessel centerline extraction accuracy and assisting in plaque assessment (115).

### 4.2 Prognostic prediction

In the field of image-guided clinical management, developing risk stratification and outcome prediction models using HR-VWI data offers a powerful pathway for translating imaging features into clinical decision support. DL technology facilitates not only the automated extraction of complex radiographic characteristics but also the integration of clinical parameters, imaging biomarkers, and temporal data, enabling the prediction of stroke recurrence, intracranial aneurysm stability, and long-term functional outcomes. By processing HR-VWI scans, DL models can quantify plaque morphology, internal heterogeneity, and enhancement patterns to achieve precise stroke risk assessment. Yang and colleagues enrolled 235 patients (132 symptomatic plaques and 103 asymptomatic plaques) and divided them into a training set (*n* = 156) and a testing set (*n* = 79). They compared three models for differentiating symptom-related intracranial and extracranial plaques: (1) the traditional model, which is based on the clinical and radiological characteristics of the plaques. This model achieved an AUC of 0.717 in the training set and 0.649 in the testing set, and the accuracy was 72.4% versus 68.4%; (2) the radiomics model was based on 1,130 radiomics features extracted from each ROI. This model achieved an AUC of 0.959 in the training set and 0.906 in the testing set, and the accuracy was 89.7% versus 83.5%; (3) five DL models (DenseNet 121, DenseNet 201, GoogleNet, ResNet 18, and ResNet 34) achieved an AUC of 0.947 in the training set and 0.956 in the testing set, the accuracy was 87.8% versus 89.9%, the sensitivity was 88.9% versus 95.2%, and the specificity was 86.4% versus 83.8%. Notably, the combination of the DL model and the traditional model achieved the best performance, with an AUC of 0.966 in the training set and 0.952 in the testing set (116).

By utilizing DL-based segmentation of VWI images from 28 patients in the USA, Guo et al. reported that this model can help identify intraplaque hemorrhage (IPH), quantify the IPH volume, and measure the effects of these methods on carotid plaque burden during long-term follow-up (117). Gao et al. enrolled 363 patients with sICAS in China and divided them into a training set (*n* = 254) and a test set (*n* = 109). A trans-CNN architecture was constructed, and its ability to predict the risk of stroke recurrence in patients with sICAS was analyzed and compared with those of two other CNNs (ResNet50 and DenseNet169). The proposed Trans-CNN model achieved an AUC of 0.951 on the training set and 0.912 on the test set. The Trans-CNN had better AUC improvement than the other two CNN models did. These results indicate that the automated detection of culprit plaque characteristics via DL can help identify lesions with a greater likelihood of causing recurrent stroke (118).

The morphological characteristics of IAs serve as important predictors of rupture risk. On the basis of 99 cases containing 120 aneurysms (91 unruptured, 29 ruptured), Ou et al. developed a backbone network of a 3D ResNet18 followed by a fully connected layer. This model achieved an AUC of 0.853, a sensitivity of 0.893, a specificity of 0.838, a PPV of 0.692, an NPV of 0.967, and an accuracy of 0.853. Further integration of clinical data with deep embedding features improved the model’s AUC to 0.853. When neurosurgeons used this system for decision support, their ability to predict rupture risk performance also improved, with AUC values increasing from 0.877 to 0.945. These results not only support the adoption of DL models in clinical settings but also present a viable framework for combining HR-VWI radiomics with DL (119).

Predicting long-term clinical outcomes on the basis of early AIS might provide essential information for its clinical management. Several studies have developed DL models for better predicting the clinical outcomes of AIS patients. Liu et al. enrolled 640 patients with AIS. They proposed a 3D ResNet model integrated with clinical variables. This fused model exhibited superior performance in predicting the 90-day modified Rankin scale (mRS) score in patients with AIS, achieving an AUC of 0.92 for poor outcomes, with significantly lower error than standalone clinical or imaging models (120). Li et al. retrospectively enrolled 363 patients with sICAS in China and collected HR-VWI images. Among those patients, 21.76% experienced stroke recurrence (79 patients), and the remaining 78.24% did not experience recurrence (284 patients). The training/validation set included 290 patients, and the test set included 73 patients. The cohort was divided into training/validation sets (*n* = 290) and a test set (*n* = 73). A combined prediction model was developed by integrating automated multiview deep feature learning and DeepSurv-based survival analysis. This model achieved a C-index of 0.872 in the internal validation set and 0.803 in the external test set; additionally, the AUCs of predictive accuracy for 1-, 2-, and 3-year recurrence rates were 0.841, 0.870, and 0.802, respectively (121). These results highlight how combining the DL with the HR-VWI enables a more personalized and accurate prognosis in patients with ischemic stroke.

### 4.3 Treatment guidance and efficacy evaluation

The management of cerebrovascular diseases has traditionally relied on empirical judgment and qualitative imaging. Currently, the integration of DL with HR-VWI is shifting this paradigm toward intelligent decision support and dynamic monitoring. For example, DL models based on HR-VWI can accurately predict IA rupture risk and stroke recurrence risk without the need for invasive procedures (119, 121). For example, Simon et al. enrolled 23 patients with AVM. HR-MRI/MRA images were collected. A DL model was developed on the basis of the 2D U-Net architecture, which achieved good classification performance for arteries, veins, brain parenchyma, and cerebrospinal fluid (Dice coefficients of 0.86, 0.91, 0.98, and 0.91, respectively) (122). In a multicenter study, Peng et al. enrolled 236 patients with 266 intracranial aneurysms (IAs) in China. The ML model (4D-Flow logistic regression, 4D-Flow-LR) and the DL model (Multicrop Attention Branch Network, MicroAB-Net) were used to predict the instability of IAs on the basis of the aneurysmal wall and aneurysm morphology-related features. 4D-Flow-LR achieved an AUC of 0.809, and MicroAB-Net achieved an AUC of 0.807. The hybrid model, which combines 4D-Flow-LR and MicroAB-Net, achieves the best performance, with an AUC value of 0.854 (123). Similarly, Tao et al. conducted a multicenter study enrolling 193 patients who underwent HR-MR-VWI examination in China. A DL model, which was named Res U-net, achieved DSCs of 0.936 (outer wall area), 0.942 (lumen area), and 0.845 (vessel wall region) on the internal test set. Robust performance was also identified in the external test set. The model achieved DSCs of 0.928 (outer wall area), 0.936 (lumen area), and 0.844 (vessel wall region) (124). Taken together, DL-based methods enable precise and consistent vessel wall segmentation in clinical HR-VWI, providing a practical approach to simplify cerebrovascular risk assessment and assist decision-making for stroke prevention and monitoring.

## 5 Clinical challenges and future directions

### 5.1 Data availability and quality bottlenecks

The primary constraint for the clinical translation of HR-VWI combined with DL lies in the scarcity of high-quality data and the difficulty of fine-grained annotation. The acquisition of HR-VWI images has high requirements for sequences, parameters, and patient cooperation. Data from different manufacturers, magnetic field strengths, and scanning protocols exhibit significant heterogeneity, which further increases the difficulty of multicenter data integration and model generalization. More importantly, the identification of vascular wall abnormalities (such as thin fibrous caps, IPH, and focal enhancement) often requires joint review by senior neuroradiologists or vascular surgeons. Subjective differences exist among different reviewers, resulting in high costs for “gold standard” annotations and challenges in establishing large-scale, highly consistent annotated databases. This highly specialized annotation process is both expensive and time-consuming, severely restricting the construction of large-scale datasets. Data imbalance is also a prominent issue. The insufficient number of samples for certain rare lesions or specific plaque types may cause the model to tend to learn the features of the majority class. In addition, legal and ethical constraints related to patient privacy and data sharing, as well as technical or compliance barriers in cross-institutional data transmission and storage, limit the establishment of multicenter, open datasets.

### 5.2 Challenges in algorithm transparency and generalization

Although DL demonstrates excellent performance in image recognition and segmentation tasks, its “black-box” nature remains one of the major obstacles to its clinical adoption. Clinicians need to understand the basis for the conclusions provided by the model during the decision-making process—especially with respect to selecting surgical or interventional treatments. The lack of interpretability weakens clinical trust and increases uncertainty regarding responsibility attribution. In addition, the generalizability and robustness of DL models are insufficient. For example, building a DL model typically depends on precise extraction of the vessel centerline for coordinate transformation; however, inaccuracies in centerline alignment can negatively affect segmentation performance. The model generally presumes that vessels have regular geometries, making it less suitable for cases involving tortuous or irregular vascular structures. This shortcoming is especially significant for certain groups—such as older adults or patients with vascular diseases—where preexisting infarcts, chronic vessel remodeling, vasculitis, or dolichoectasia can lead to atypical vascular anatomy. Future research that adopts adaptive transformation methods may help overcome these issues and enhance the model’s generalizability for complex vascular anatomies (124).

Furthermore, the model’s sensitivity to fluctuations in image quality, artifacts, and noise also poses challenges. In real clinical settings, image quality degradation caused by various factors (e.g., patient motion, equipment calibration errors) can affect the stable performance of the model. Limitations may also exist in the model architecture itself. Many existing methods fail to fully exploit the 3D spatial information contained in HR-VWI data and the complementary information among multicontrast sequences, which restricts the model’s ability to characterize complex vascular lesions. If these algorithm-level flaws are not addressed, they severely hinder the reliable application of DL in real clinical environments.

Insufficient data in the training, validation, or test sets may limit the global deployment of DL models. The sizes (or number of cases) in the training set are often determined on the basis of multichannel images for tissue segmentation. Many factors, such as the type of tissue and the acquisition protocol, can affect the minimum training size. Without the minimum training size needed for accurate analysis, the results may not be completely generalizable.

### 5.3 Barriers to clinical integration and validation

The integration of DL and HR-VWI technologies from research platforms to clinical tools faces multidimensional institutional and practical barriers. At this stage, the vast majority of studies are retrospective or single-center validations, lacking large-scale prospective, multicenter clinical trials to demonstrate their actual value in improving the clinical outcomes of patients from different hospitals or countries. However, regulatory authorities and hospital procurement decisions often rely on such high-quality evidence. Regulatory approval and the lack of standardization also represent important barriers. Currently, there is no clear regulatory pathway for approving DL-based HR-VWI analysis tools as medical devices. Moreover, the industry lacks unified technical standards and evaluation norms, making it difficult for these tools to enter clinical use in a legal and compliant manner. In addition, seamlessly integrating DL tools into existing radiology workflows and hospital information systems—without increasing the workload of clinicians—involves issues such as interoperability between IT systems (e.g., PACS, RIS, and electronic medical records) and DL systems, data interface specifications, real-time performance requirements, and information security. Furthermore, clinicians’ acceptance and willingness to use these tools are key factors. Clinicians may adopt a cautious attitude toward new technologies or lack sufficient training to correctly understand and apply the output results of these tools. Cost-effectiveness considerations also cannot be ignored. Deploying and maintaining DL systems requires considerable resource investment, and whether they can lead to corresponding improvements in clinical benefits and economic value still needs further verification. Therefore, promoting clinical translation requires collaborative efforts in evidence generation, regulatory compliance, information system integration, user training, and economic evaluation.

### 5.4 Technology integration and trial strategies for precision medicine

Advancing clinically useful HR-VWI, ML, and DL models will require progress on both the technical and study design fronts. Technically, priority should be given to building multimodal frameworks that combine HR-VWI with perfusion imaging, hemodynamics, and clinical data to improve pathological assessment and risk prediction. Approaches such as self-supervised pretraining, semisupervised learning, and federated learning can strengthen model generalizability on limited or diverse datasets while safeguarding privacy. Interpretability research should advance alongside model performance, with visual, traceable outputs for key features such as plaque enhancement or intraplaque hemorrhage. Additionally, unified evaluation indicators, benchmark datasets, and open challenges should be established to facilitate objective comparisons of different models and methods. For clinical deployment, attention should also be given to the development of continuous performance monitoring mechanisms, model retraining strategies, and regulatory compliance pathways. This creates a closed-loop framework spanning technical development, clinical validation, and regulatory approval. The long-term objective is a multimodal precision medicine ecosystem built around HR-VWI and powered by DL—one that enables individualized cerebrovascular disease management while maintaining safety and clinical interpretability.

### 5.5 Implications of population diversity and biological heterogeneity

Population diversity, which encompasses demographic, ethnic, geographic, and socioeconomic variations, is a core factor that shapes the generalizability, accuracy, and clinical translation of DL and HR-VWI in the assessment of various intracranial vascular lesions. DL models for cerebral vascular image analysis are predominantly trained on homogeneous or hospital-specific cohorts, which lack representation of the full spectrum of population diversity. This leads to three major clinical limitations: (1) Reduced performance in underrepresented populations. Ethnic/racial variations in cerebral vascular anatomical features (e.g., MMD and cerebrovascular atherosclerosis incidence, arterial calibre, and intracranial artery tortuosity), as well as geographic differences in disease risk factors (e.g., hypertension, diabetes, and smoking prevalence), cause DL models to exhibit lower accuracy and higher false positive/negative rates when applied to non-Caucasian, low-resource region, or elderly/pediatric cohorts. For example, models trained on Asian populations may fail to detect small vessel occlusion accurately in European stroke patients with distinct vascular morphologies. (2) Exacerbation of clinical diagnostic disparities. Socioeconomic diversity further amplifies this issue—populations with limited access to high-quality imaging or early screening have underrepresented data in training sets, leading DL models to perform poorly for these groups and widening gaps in diagnostic equity for cerebral vascular diseases across different socioeconomic strata; (3) population diversity also includes institutional/scanner diversity (a practical subset in clinical imaging), where variations in imaging equipment, postprocessing workflows, and radiologist annotation standards across hospitals create a “domain shift”—a direct consequence of population diversity in data collection—and render model performance inconsistent across clinical centers.

Biological heterogeneity also has a great impact on the clinical application of DL models. Cerebral vascular diseases exhibit extreme biological heterogeneity: (1) Interindividual differences in disease etiology. e.g., ischemic stroke from cardioembolism vs. large artery atherosclerosis; (2) pathological manifestations. e.g., aneurysm size, location, and wall enhancement; (3) intraindividual changes in disease progression. e.g., stroke lesion evolution and posttreatment vascular remodeling; (4) comorbidity profiles. e.g., cerebral vascular disease combined with dementia, heart disease, or renal failure.

These heterogeneities pose fundamental challenges to DL models: (1) Degraded performance in complex pathological scenarios. Since DL models rely on learned patterns from labeled disease features, biological heterogeneity leads to feature variability (such as diverse stroke lesion locations/sizes and atypical aneurysm morphologies) that may fall outside the distribution of training data. This results in model uncertainty or misclassification for rare or complex cerebral vascular pathologies, reducing the reliability of automated diagnosis and outcome prediction. (2) Limited validity of personalized outcome prediction. A core clinical application of DL in cerebral vascular disease is for predicting treatment response and clinical outcomes. Biological heterogeneity in individual physiological responses to treatment (e.g., varying antiplatelet drug efficacy, different rates of vascular repair) means that DL models trained on aggregate population data fail to capture individual-specific biological traits, leading to inaccurate personalized predictions and limiting the model’s value for clinical decision-making. (3) Intraindividual temporal heterogeneity. There are dynamic changes in cerebral vascular pathology over time, such as from acute stroke lesion expansion to chronic small vessel disease progression. The process requires DL models to adapt to temporal feature shifts. However, static training data (cross-sectional imaging) cannot capture this heterogeneity, resulting in poor long-term outcome prediction performance.

Population diversity and biological heterogeneity do not act independently—their synergy further complicates the clinical application of DL models. For example, a specific ethnic population (population diversity) may have a greater prevalence of a rare pathological subtype of cerebral vascular disease (biological heterogeneity); the combined underrepresentation of this group in training data leads to severe model underperformance that cannot be attributed to either factor alone. Additionally, population-level differences in risk factor exposure (e.g., high hypertension prevalence in a certain geographic region) drive biological heterogeneity in disease severity and manifestation, creating a feedback loop that deepens the mismatch between DL models and real-world clinical populations. This synergy makes it difficult to isolate and address model limitations, significantly hindering the seamless translation of DL-based image analysis from the laboratory to clinical practice.

Despite being major challenges, population diversity and biological heterogeneity act as critical drivers for advancing DL-based HR-VWI analysis. There are several potential strategies for overcoming such challenges: (1) Demand for diverse and representative training datasets. The limitations imposed by population diversity have spurred global efforts to construct multicenter, multiethnic, and cross-geographic imaging datasets for cerebral vascular diseases, including standardized annotation and harmonization of imaging protocols to reduce domain shifts. (2) Development of heterogeneity-aware DL models. To address biological heterogeneity, advanced DL architectures (e.g., attention mechanisms, graph neural networks, and multimodal fusion models) can capture complex, heterogeneous pathological features and individual-specific biological traits. Additionally, personalized DL models (e.g., transfer learning, few-shot learning, and federated learning) are being developed to adapt to rare disease subtypes and intraindividual temporal changes, improving the accuracy of diagnosis and outcome prediction for heterogeneous clinical cases. (3) Integration of clinical and biological metadata. Population diversity and biological heterogeneity highlight the need to move beyond pure image-based DL models to integrate multimodal data (e.g., clinical demographics, genetic information, blood biomarkers, and lifestyle factors). This integration enables models to account for population-level variations and individual biological heterogeneity, enhancing the interpretability and predictive validity of DL-based analysis for clinical decision-making.

## 6 Conclusion

The combination of DL and HR-VWI has significantly advanced the clinical management of intracranial vascular lesions, shifting the focus from technical refinement to precision medicine. HR-VWI allows direct visualization of the intracranial vessel wall and detection of subtle pathological changes, whereas DL algorithms provide efficient, scalable quantitative analysis—turning lesion identification, risk assessment, and treatment evaluation from subjective judgment into objective, data-informed decisions. Studies have confirmed that DL models perform strongly in detecting atherosclerosis, segmenting vessel walls, identifying culprit plaques, predicting stroke recurrence, and assessing aneurysm risk. By integrating radiomic features with clinical variables, these models also support more personalized prognostic predictions.

However, most current studies have been performed with single or limited cohorts of clinical cases. The DL models depend on the combination of semiautomated segmentation and expert evaluation. Population diversity and biological heterogeneity, which are fundamental, unavoidable factors in the clinical application of DL-based cerebral vascular disease image analysis, may often be ignored; these factors act as the primary barriers to achieving consistent, equitable, and reliable model performance across real-world clinical populations. Finally, they limit the model’s ability to deliver personalized diagnosis and outcome prediction for heterogeneous disease cases. These challenges also serve as pivotal catalysts for the evolution of the field, driving the development of diverse, standardized datasets, heterogeneity-aware DL architectures, multimodal data integration, and adaptive clinical deployment strategies. Future development should focus on improving the interpretability and clinical applicability of DL models.

## Glossary

AI: artificial intelligence
ASMR: Age-standardized mortality rate
AVM: arteriovenous malformation
AUC: area under the curve
AWE: IA wall enhancement
CCAD: Cervicocranial arterial dissection
CICAO: chronic internal carotid artery occlusion
CMRA: coronary magnetic resonance angiography
CNN: Convolutional Neural Networks
CRNN: convolutional recurrent neural network
CS-MRI: compressed sensing magnetic resonance imaging
CNSV: central nervous system vasculitis
CTA: computed tomography angiography
CTP: computed tomography perfusion
CVD: cerebrovascular disease
DWI: diffusion-weighted imaging
DL: Deep Learning
DSA: digital subtraction angiography
FVS: fast virtual stenting
GBD: Global Burden of Disease
HR-VWI: high-resolution magnetic resonance vessel wall imaging
ICAD: Intracranial atherosclerotic disease
ICAO: Intracranial arterial occlusion
IA: Intracranial aneurysm;
UIA: Unruptured IA
KNN: K-nearest neighbor
LDA: linear discriminant analysis
LSTM: long short-term memory
LMVV: Large and Medium-sized Vessel Vasculopathy
ML: Machine Learning
MLP: multilayer perceptron
MMD: Moyamoya disease
MRA: magnetic resonance angiography
Mrs.: modified Rankin scale;
NCCT: noncontrast computed tomography
PA: parent artery
RF: random forest
RNN: Recurrent Neural Network
SVM: Support Vector Machine
SVV: small vessel vasculopathy
TEVAR: Thoracic endovascular aortic repair
TRI: Texture Representation of Images
VBDAs: Vertebrobasilar dissecting aneurysms
VWE: Vascular wall enhancement;

## References

[1] GBD 2021 Stroke Risk Factor Collaborators. Global, regional, and national burden of stroke and its risk factors, 1990-2021: a systematic analysis for the global burden of disease study 2021. Lancet Neurol. (2024) 23:973–1003. doi: 10.1016/S1474-4422(24)00369-7, 39304265 PMC12254192

[2] Zhu W He X Huang D Jiang Y Hong W Ke S . Global and regional burden of ischemic stroke disease from 1990 to 2021: an age-period-cohort analysis. Transl Stroke Res. (2025) 16:1474–85. doi: 10.1007/s12975-024-01319-9 39699770

[3] Li XY Kong XM Yang CH Cheng ZF Lv JJ Guo H . Global, regional, and national burden of ischemic stroke, 1990-2021: an analysis of data from the global burden of disease study 2021. EClinicalMedicine. (2024) 75:102758. doi: 10.1016/j.eclinm.2024.102758, 39157811 PMC11327951

[4] Panagiotopoulos E Stefanou MI Magoufis G Safouris A Kargiotis O Psychogios K . Prevalence, diagnosis and management of intracranial atherosclerosis in white populations: a narrative review. Neurol Res Pract. (2024) 6:54. doi: 10.1186/s42466-024-00341-4, 39523357 PMC11552123

[5] Ito T Fujiyoshi A Ohkubo T Shiino A Shitara S Miyagawa N . Asymptomatic intracranial vascular lesions and cognitive function in a general population of Japanese men: Shiga epidemiological study of subclinical atherosclerosis (SESSA). Cerebrovasc Dis. (2025) 54:1–11. doi: 10.1159/000546882, 40505633 PMC12279311

[6] Wang J Lu J Qi P Li C Yang X Chen K . Association between kinking of the cervical carotid or vertebral artery and ischemic stroke/TIA. Front Neurol. (2022) 13:1008328. doi: 10.3389/fneur.2022.1008328, 36176562 PMC9513150

[7] Sousa JA Sondermann A Bernardo-Castro S Varela R Donato H Sargento-Freitas J. CTA and CTP for detecting distal medium vessel occlusions: a systematic review and meta-analysis. AJNR Am J Neuroradiol. (2023) 45:51–6. doi: 10.3174/ajnr.A8080, 38164544 PMC10756569

[8] Keil F Bergkemper A Birkhold A Kowarschik M Tritt S Berkefeld J. 4D flat panel Conebeam CTA for analysis of the Angioarchitecture of cerebral AVMs with a novel software prototype. AJNR Am J Neuroradiol. (2022) 43:102–9. doi: 10.3174/ajnr.A7382, 35027345 PMC8757557

[9] Mossa-Basha M Alexander M Gaddikeri S Yuan C Gandhi D. Vessel wall imaging for intracranial vascular disease evaluation. J Neurointerv Surg. (2016) 8:1154–9. doi: 10.1136/neurintsurg-2015-012127, 26769729 PMC5484417

[10] Griffin WF Choi AD Riess JS Marques H Chang HJ Choi JH . AI evaluation of stenosis on coronary CTA, comparison with quantitative coronary angiography and fractional flow reserve: a CREDENCE trial substudy. JACC Cardiovasc Imaging. (2023) 16:193–205. doi: 10.1016/j.jcmg.2021.10.020, 35183478

[11] Nurmohamed NS van Rosendael AR Danad I Ngo-Metzger Q Taub PR Ray KK . Atherosclerosis evaluation and cardiovascular risk estimation using coronary computed tomography angiography. Eur Heart J. (2024) 45:1783–800. doi: 10.1093/eurheartj/ehae190, 38606889 PMC11129796

[12] Patil S Patel D Kata R Teichner E Subtirelu R Ayubcha C . Molecular imaging with PET in the assessment of vascular dementia and cerebrovascular disease. PET Clin. (2025) 20:121–31. doi: 10.1016/j.cpet.2024.09.001, 39477719

[13] Schulze K Stantien AM Williams MC Vassiliou VS Giannopoulos AA Nieman K . Coronary CT angiography evaluation with artificial intelligence for individualized medical treatment of atherosclerosis: a consensus statement from the QCI study group. Nat Rev Cardiol. (2025) 23:100–15. doi: 10.1038/s41569-025-01191-6, 40751112

[14] Vranic JE Hartman JB Mossa-Basha M. High-resolution magnetic resonance Vessel Wall imaging for the evaluation of intracranial vascular pathology. Neuroimaging Clin N Am. (2021) 31:223–33. doi: 10.1016/j.nic.2021.01.005, 33902876

[15] Chen L Guo Q Zhao J Bao H Meng F Meng L. Evaluation of risk factors for acute stroke using combined CTA and MR HR-VWI imaging. Front Neurol. (2025) 16:1551682. doi: 10.3389/fneur.2025.1551682, 40904822 PMC12401676

[16] Chen T Zhu W Bai X Mossa-Basha M Zhao Y Pei X . A radiomic model based on 7T intracranial vessel wall imaging for identification of culprit middle cerebral artery plaque associated with subcortical infarctions. J Cardiovasc Magn Reson. (2025) 27:101956. doi: 10.1016/j.jocmr.2025.101956, 40939694 PMC12730851

[17] Guo Y Yuan C Su Y Wang Z Li S Wang B . Predicting periprocedural complications risk in intracranial angioplasty and stenting from integrated high-resolution vessel wall imaging radiomics and clinical characteristics. Neuroradiology. (2025) 67:2459–69. doi: 10.1007/s00234-025-03757-0, 40920202

[18] Hou Y Ren L Cao C Zhang H Zhao W Zhu J . The additional value of high-resolution vessel wall imaging in screening suitable chronic internal carotid artery occlusion candidates for endovascular recanalization: comparison with digital subtraction angiography. Acta Radiol. (2023) 64:1702–11. doi: 10.1177/02841851221127563 36148918

[19] Kulkarni SV Poornapushpakala S. Multi-scale based network and adaptive EfficientnetB7 with ASPP: analysis of novel brain tumor segmentation and classification. Curr Med Imag. (2025) 21:e15734056419990. doi: 10.2174/0115734056419990250904093436 PMC13223442 40965060

[20] Liu G Li Q Wang Y Zhou X Li Y Liu Y . Resolution enhancement and target segmentation of medical images based on the frequency-domain information in deep learning. Appl Opt. (2025) 64:7083–92. doi: 10.1364/AO.557903, 40981883

[21] Osman YBM Li C Elsayed N Diakite A Wang S Wang S. Enhancing semi-supervised learning for fine-grained 3D cerebrovascular segmentation with cross-consistency and uncertainty estimation. Med Phys. (2025) 52:e70017. doi: 10.1002/mp.70017, 40985655

[22] Ryu WS Schellingerhout D Lee H Lee KJ Kim CK Kim BJ . Deep learning-based automatic classification of ischemic stroke subtype using diffusion-weighted images. J Stroke. (2024) 26:300–11. doi: 10.5853/jos.2024.00535, 38836277 PMC11164582

[23] Marcus A Mair G Chen L Hallett C Cuervas-Mons CG Roi D . Deep learning biomarker of chronometric and biological ischemic stroke lesion age from unenhanced CT. NPJ Digit Med. (2024) 7:338. doi: 10.1038/s41746-024-01325-z, 39643604 PMC11624201

[24] Sun J Ju GL Qu YH Xie HH Sun HX Han SY . Deep learning for segmenting ischemic stroke infarction in non-contrast CT scans by utilizing asymmetry. Clin Neuroradiol. (2025). doi: 10.1007/s00062-025-01559-8, 40908314

[25] Zhang S Zhuang Y Luo Y Zhu F Zhao W Zeng H. Deep learning-based automated lesion segmentation on pediatric focal cortical dysplasia II preoperative MRI: a reliable approach. Insights Imaging. (2024) 15:71. doi: 10.1186/s13244-024-01635-6, 38472513 PMC10933224

[26] Chandrashekar A Handa A Shivakumar N Lapolla P Uberoi R Grau V . A deep learning pipeline to Automate high-resolution arterial segmentation with or without intravenous contrast. Ann Surg. (2022) 276:e1017–lpagee1027. doi: 10.1097/SLA.0000000000004595, 33234786 PMC9645535

[27] Pias AD Pereira-Macedo J Marreiros A António N Rocha-Neves J. Advancing vascular surgery: the role of artificial intelligence and machine learning in managing carotid stenosis. Port J Card Thorac Vasc Surg. (2024) 31:55–64. doi: 10.48729/pjctvs.411, 39820882

[28] Chen X Lei Y Su J Yang H Ni W Yu J . A review of artificial intelligence in cerebrovascular disease imaging: applications and challenges. Curr Neuropharmacol. (2022) 20:1359–82. doi: 10.2174/1570159X19666211108141446, 34749621 PMC9881077

[29] Coelho A Peixoto J Mansilha A. Potential impact of artificial intelligence in predicting cerebrovascular events in patients with carotid artery stenosis. Int Angiol. (2025) 44:189–94. doi: 10.23736/S0392-9590.25.05424-0, 40856782

[30] Zhao T Lin G Chen W Wu J Hu W Xu L . Predicting symptomatic carotid artery plaques with radiomics-based carotid perivascular adipose tissue characteristics: a multicenter, multiclassifier study. BMC Med Imaging. (2025) 25:337. doi: 10.1186/s12880-025-01876-x, 40830841 PMC12363075

[31] Liu Y Yuan K Zou L Lei C Xu R He S . Combining machine learning with external validation to explore necroptosis and immune response in moyamoya disease. BMC Immunol. (2025) 26:6. doi: 10.1186/s12865-025-00686-8, 39948449 PMC11823220

[32] Momeni S Fazlollahi A Yates P Rowe C Gao Y Liew AW . Synthetic microbleeds generation for classifier training without ground truth. Comput Methods Prog Biomed. (2021) 207:106127. doi: 10.1016/j.cmpb.2021.106127, 34051412

[33] Tsuneki M. Deep learning models in medical image analysis. J Oral Biosci. (2022) 64:312–20. doi: 10.1016/j.job.2022.03.003 35306172

[34] Wang Y Liu L Wang C. Trends in using deep learning algorithms in biomedical prediction systems. Front Neurosci. (2023) 17:1256351. doi: 10.3389/fnins.2023.1256351, 38027475 PMC10665494

[35] Hong W Kang J Kim SE Jeong T Yoon CJ Lee IJ . Deep learning-based diagnosis of Femoropopliteal artery Steno-occlusion using maximum intensity projection images of CT angiography. Tomography. (2025) 11:104. doi: 10.3390/tomography11090104, 41003487 PMC12473302

[36] Dai D Dong C Huang H Liu F Li Z Xu S. Improving the performance of medical image segmentation with instructive feature learning. Med Image Anal. (2026) 107:103818. doi: 10.1016/j.media.2025.103818, 41005261

[37] Chen X Wang X Zhang K Fung KM Thai TC Moore K . Recent advances and clinical applications of deep learning in medical image analysis. Med Image Anal. (2022) 79:102444. doi: 10.1016/j.media.2022.102444, 35472844 PMC9156578

[38] Chen W Huang H Huang J Wang K Qin H Wong KKL. Deep learning-based medical image segmentation of the aorta using XR-MSF-U-net. Comput Methods Prog Biomed. (2022) 225:107073. doi: 10.1016/j.cmpb.2022.107073, 36029551

[39] Zhu Y Liu Y Zhou X. Automated segmentation of retinal vessel using HarDNet fully convolutional networks. PLoS One. (2025) 20:e0330641. doi: 10.1371/journal.pone.0330641, 40920773 PMC12416673

[40] Quintana-Quintana OJ De León-Cuevas A González-Gutiérrez A Gorrostieta-Hurtado E Tovar-Arriaga S. Dual U-net-based conditional generative adversarial network for blood vessel segmentation with reduced cerebral MR training volumes. Micromachines. (2022) 13:823. doi: 10.3390/mi13060823, 35744437 PMC9229670

[41] Bai Y Li D Duan Q Chen X. Analysis of high-resolution reconstruction of medical images based on deep convolutional neural networks in lung cancer diagnostics. Comput Methods Prog Biomed. (2022) 217:106592. doi: 10.1016/j.cmpb.2021.106592, 35172253

[42] Chatterjee S Prabhu K Pattadkal M Bortsova G Sarasaen C Dubost F . DS6, deformation-aware semi-supervised learning: application to small vessel segmentation with Noisy training data. J Imaging. (2022) 8:259. doi: 10.3390/jimaging8100259, 36286353 PMC9605070

[43] Lin K Jie B Dong P Ding X Bian W Liu M. Convolutional recurrent neural network for dynamic functional MRI analysis and brain disease identification. Front Neurosci. (2022) 16:933660. doi: 10.3389/fnins.2022.933660, 35873806 PMC9298744

[44] Mehdipour Ghazi M Nielsen M Pai A Cardoso MJ Modat M Ourselin S . Training recurrent neural networks robust to incomplete data: application to Alzheimer's disease progression modeling. Med Image Anal. (2019) 53:39–46. doi: 10.1016/j.media.2019.01.004, 30682584

[45] Eun DI Jang R Ha WS Lee H Jung SC Kim N. Deep-learning-based image quality enhancement of compressed sensing magnetic resonance imaging of vessel wall: comparison of self-supervised and unsupervised approaches. Sci Rep. (2020) 10:13950. doi: 10.1038/s41598-020-69932-w, 32811848 PMC7434911

[46] Bathla G Messina SA Black DF Benson JC Kollasch P Nickel MD . Deep learning-based reconstruction of 3D T1 SPACE Vessel Wall imaging provides improved image quality with reduced scan times: a preliminary study. AJNR Am J Neuroradiol. (2024) 45:1655–60. doi: 10.3174/ajnr.A8382, 38889969 PMC11543076

[47] Ota H Morita Y Vucevic D Higuchi S Takagi H Kutsuna H . Motion robust coronary MR angiography using zigzag centric ky-kz trajectory and high-resolution deep learning reconstruction. MAGMA. (2024) 37:1105–17. doi: 10.1007/s10334-024-01172-9, 38916681

[48] Livne M Rieger J Aydin OU Taha AA Akay EM Kossen T . A U-net deep learning framework for high performance vessel segmentation in patients with cerebrovascular disease. Front Neurosci. (2019) 13:97. doi: 10.3389/fnins.2019.00097, 30872986 PMC6403177

[49] Shariaty F Mohebi M Barzegar-Golmoghani E Pavlov V Sarmi SHA Behzadpour MH . Deep vessel segmentation with U-net and texture representation of image (TRI) features provides a foundation for improved objective and automated analysis of coronary artery disease from angiography. Comput Methods Prog Biomed. (2025) 272:109072. doi: 10.1016/j.cmpb.2025.109072, 40983000

[50] Zhou H Xiao J Li D Fan Z Ruan D. Intracranial vessel wall segmentation with deep learning using a novel tiered loss function incorporating class inclusion. Med Phys. (2022) 49:6975–85. doi: 10.1002/mp.15860, 35815927 PMC9742123

[51] Yang X Yu P Zhang H Zhang R Liu Y Li H . Deep learning algorithm enables cerebral venous thrombosis detection with routine brain magnetic resonance imaging. Stroke. (2023) 54:1357–66. doi: 10.1161/STROKEAHA.122.041520, 36912139

[52] Cetinoglu YK Koska IO Uluc ME Gelal MF. Detection and vascular territorial classification of stroke on diffusion-weighted MRI by deep learning. Eur J Radiol. (2021) 145:110050. doi: 10.1016/j.ejrad.2021.110050, 34839210

[53] Lu J Zhou Y Lv W Zhu H Tian T Yan S . Identification of early invisible acute ischemic stroke in non-contrast computed tomography using two-stage deep-learning model. Theranostics. (2022) 12:5564–73. doi: 10.7150/thno.74125, 35910809 PMC9330528

[54] Wang C Shi Z Yang M Huang L Fang W Jiang L . Deep learning-based identification of acute ischemic core and deficit from non-contrast CT and CTA. J Cereb Blood Flow Metab. (2021) 41:3028–38. doi: 10.1177/0271678X211023660, 34102912 PMC8756471

[55] Chen G Ru J Zhou Y Rekik I Pan Z Liu X . MTANS: multi-scale mean teacher combined adversarial network with shape-aware embedding for semi-supervised brain lesion segmentation. NeuroImage. (2021) 244:118568. doi: 10.1016/j.neuroimage.2021.118568, 34508895

[56] Wang G Song T Dong Q Cui M Huang N Zhang S. Automatic ischemic stroke lesion segmentation from computed tomography perfusion images by image synthesis and attention-based deep neural networks. Med Image Anal. (2020) 65:101787. doi: 10.1016/j.media.2020.101787, 32712524

[57] Fan F Song H Jiang J He H Sun D Xu Z . Development and validation of a multimodal deep learning framework for vascular cognitive impairment diagnosis. iScience. (2024) 27:110945. doi: 10.1016/j.isci.2024.110945, 39391736 PMC11465129

[58] Deshpande A Elliott J Jiang B Tahsili-Fahadan P Kidwell C Wintermark M . End to end stroke triage using cerebrovascular morphology and machine learning. Front Neurol. (2023) 14:1217796. doi: 10.3389/fneur.2023.1217796, 37941573 PMC10628321

[59] Wouters A Robben D Christensen S Marquering HA Roos YBWEM van Oostenbrugge R . Prediction of stroke infarct growth rates by baseline perfusion imaging. Stroke. (2022) 53:569–77. doi: 10.1161/STROKEAHA.121.034444, 34587794 PMC8792202

[60] Yu Y Xie Y Thamm T Gong E Ouyang J Huang C . Use of deep learning to predict final ischemic stroke lesions from initial magnetic resonance imaging. JAMA Netw Open. (2020) 3:e200772. doi: 10.1001/jamanetworkopen.2020.0772, 32163165 PMC7068232

[61] Nishi H Oishi N Ishii A Ono I Ogura T Sunohara T . Deep learning-derived high-level neuroimaging features predict clinical outcomes for large vessel occlusion. Stroke. (2020) 51:1484–92. doi: 10.1161/STROKEAHA.119.028101, 32248769

[62] Song JW Pavlou A Xiao J Kasner SE Fan Z Messé SR. Vessel Wall magnetic resonance imaging biomarkers of symptomatic intracranial atherosclerosis: a Meta-analysis. Stroke. (2021) 52:193–202. doi: 10.1161/STROKEAHA.120.031480, 33370193 PMC7773134

[63] Corti A De Paolis A Grossman P Dinh PA Aikawa E Weinbaum S . The effect of plaque morphology, material composition and microcalcifications on the risk of cap rupture: a structural analysis of vulnerable atherosclerotic plaques. Front Cardiovasc Med. (2022) 9:1019917. doi: 10.3389/fcvm.2022.1019917, 36277774 PMC9583261

[64] Bigeh A Shekar C Gulati M. Sex differences in coronary artery calcium and long-term CV mortality. Curr Cardiol Rep. (2020) 22:21. doi: 10.1007/s11886-020-1267-9, 32052199

[65] Sun J Underhill HR Hippe DS Xue Y Yuan C Hatsukami TS. Sustained acceleration in carotid atherosclerotic plaque progression with intraplaque hemorrhage: a long-term time course study. JACC Cardiovasc Imaging. (2012) 5:798–804. doi: 10.1016/j.jcmg.2012.03.014, 22897993 PMC3422506

[66] Liu Y Wang M Zhang B Wang W Xu Y Han Y . Size of carotid artery intraplaque hemorrhage and acute ischemic stroke: a cardiovascular magnetic resonance Chinese atherosclerosis risk evaluation study. J Cardiovasc Magn Reson. (2019) 21:36. doi: 10.1186/s12968-019-0548-1, 31262337 PMC6604180

[67] Pu Y Dou X Liu L. Natural history of intracranial atherosclerotic disease. Front Neurol. (2014) 5:125. doi: 10.3389/fneur.2014.00125, 25071710 PMC4091030

[68] Qiao Y Suri FK Zhang Y Liu L Gottesman R Alonso A . Racial differences in prevalence and risk for intracranial atherosclerosis in a US Community-based population. JAMA Cardiol. (2017) 2:1341–8. doi: 10.1001/jamacardio.2017.4041, 29094154 PMC5814999

[69] Kim JS Kim YJ Ahn SH Kim BJ. Location of cerebral atherosclerosis: why is there a difference between east and west? Int J Stroke. (2018) 13:35–46. doi: 10.1177/1747493016647736, 27145795

[70] Kurosaki Y Yoshida K Fukumitsu R Sadamasa N Handa A Chin M . Carotid artery plaque assessment using quantitative expansive remodeling evaluation and MRI plaque signal intensity. J Neurosurg. (2016) 124:736–42. doi: 10.3171/2015.2.JNS142783, 26361279

[71] Xu WH Li ML Gao S Ni J Zhou LX Yao M . Plaque distribution of stenotic middle cerebral artery and its clinical relevance. Stroke. (2011) 42:2957–9. doi: 10.1161/STROKEAHA.111.618132, 21799160

[72] Lu SS Ge S Su CQ Xie J Shi HB Hong XN. Plaque distribution and characteristics in low-grade middle cerebral artery stenosis and its clinical relevance: a 3-dimensional high-resolution magnetic resonance imaging study. J Stroke Cerebrovasc Dis. (2018) 27:2243–9. doi: 10.1016/j.jstrokecerebrovasdis.2018.04.010, 29752069

[73] Shi Z Li J Zhao M Zhang X Degnan AJ Mossa‐Basha M . Progression of plaque burden of intracranial atherosclerotic plaque predicts recurrent stroke/transient ischemic attack: a pilot follow-up study using higher-resolution MRI. J Magn Reson Imaging. (2021) 54:560–70. doi: 10.1002/jmri.27561, 33600033 PMC8359205

[74] Yang WJ Abrigo J Soo YO Soo YO-Y Wong S Wong K-S . Regression of plaque enhancement within symptomatic middle cerebral artery atherosclerosis: a high-resolution MRI study. Front Neurol. (2020) 11:755. doi: 10.3389/fneur.2020.00755, 32849214 PMC7399098

[75] Fakih R Roa JA Bathla G Olalde H Varon A Ortega-Gutierrez S . Detection and quantification of symptomatic atherosclerotic plaques with high-resolution imaging in cryptogenic stroke. Stroke. (2020) 51:3623–31. doi: 10.1161/STROKEAHA.120.031167, 32998652

[76] Zhou Z Chen S Balu N Chu B Zhao X Sun J . Neural network enhanced 3D turbo spin echo for MR intracranial vessel wall imaging. Magn Reson Imaging. (2021) 78:7–17. doi: 10.1016/j.mri.2021.01.004, 33548457 PMC7979503

[77] Wu J Xin J Yang X Sun J Xu D Zheng N . Deep morphology aided diagnosis network for segmentation of carotid artery vessel wall and diagnosis of carotid atherosclerosis on black-blood vessel wall MRI. Med Phys. (2019) 46:5544–61. doi: 10.1002/mp.13739, 31356693

[78] Ma Y Wang M Qiao Y Wen Y Zhu Y Jiang K . Feasibility of artificial intelligence constrained compressed SENSE accelerated 3D isotropic T1 VISTA sequence for Vessel Wall MR imaging: exploring the potential of higher acceleration factors compared to traditional compressed SENSE. Acad Radiol. (2024) 31:3971–81. doi: 10.1016/j.acra.2024.03.041, 38664146

[79] Wang J Yu F Zhang M Lu J Qian Z. A 3D framework for segmentation of carotid artery vessel wall and identification of plaque compositions in multi-sequence MR images. Comput Med Imaging Graph. (2024) 116:102402. doi: 10.1016/j.compmedimag.2024.102402, 38810486

[80] Lv Y Ma X Zhao W Ju J Yan P Li S . Association of plaque characteristics with long-term stroke recurrence in patients with intracranial atherosclerotic disease: a 3D high-resolution MRI-based cohort study. Eur Radiol. (2024) 34:3022–31. doi: 10.1007/s00330-023-10278-y, 37870623 PMC11126465

[81] Shao S Wang T Zhu L Gao Y Fan X Lu Y . Correlation of intracranial and extracranial carotid atherosclerotic plaque characteristics with ischemic stroke recurrence: a high-resolution vessel wall imaging study. Front Neurol. (2025) 15:1514711. doi: 10.3389/fneur.2024.1514711, 39882374 PMC11774726

[82] Tian X Shi Z Wang Z Xu B Peng WJ Zhang XF . Characteristics of culprit intracranial plaque without substantial stenosis in ischemic stroke using three-dimensional high-resolution vessel wall magnetic resonance imaging. Front Neurosci. (2023) 17:1160018. Published 2023 Mar 23. doi: 10.3389/fnins.2023.1160018, 37034175 PMC10076565

[83] Seo WK Oh K Suh SI Seol HY. Clinical significance of wall changes after recanalization therapy in acute stroke: high-resolution Vessel Wall imaging. Stroke. (2017) 48:1077–80. doi: 10.1161/STROKEAHA.116.015429, 28258254

[84] Chao L Qingbin M Haowen X Shanshan X Qichang F Zhen C . Imaging predictors for endovascular recanalization of non-acute occlusion of internal carotid artery based on 3D T1-SPACE MRI and DSA. Front Neurol. (2021) 12:692128. Published 2021 Oct 26. doi: 10.3389/fneur.2021.692128, 34764924 PMC8576573

[85] Hou Z Yan L Zhang Z Jing J Lyu J Hui FK High-resolution magnetic resonance vessel wall imaging-guided endovascular recanalization for nonacute intracranial artery occlusion. J Neurosurg 2021;137:412–418. Published 2021 Dec 3. doi: 10.3171/2021.9.JNS211770 34861645

[86] Chai S Sheng Z Xie W Wang C Liu S Tang R . Assessment of apparent internal carotid tandem occlusion on high-resolution Vessel Wall imaging: comparison with digital subtraction angiography. AJNR Am J Neuroradiol. (2020) 41:693–9. doi: 10.3174/ajnr.A6452, 32115423 PMC7144637

[87] Huang CH Li Q Wen L Wang GX Zhang D. Association of wall enhancement on high-resolution magnetic resonance imaging with morphology and hemodynamics in unruptured intracranial aneurysms. Neurol Res. (2025) 47:710–22. doi: 10.1080/01616412.2025.2497482, 40314243

[88] Roa JA Zanaty M Ishii D Lu Y Kung DK Starke RM Decreased contrast enhancement on high-resolution vessel wall imaging of unruptured intracranial aneurysms in patients taking aspirin. J Neurosurg 2020;134:902–908. Published 2020 Mar 6. doi:doi: 10.3171/2019.12.JNS193023 32114538 PMC7483906

[89] Leber SL Hassler EM Michenthaler M Renner W Deutschmann H Reishofer G. Wall enhancement of coiled intracranial aneurysms is associated with aneurysm recanalization: a cross-sectional study. AJNR Am J Neuroradiol. (2024) 45:599–604. Published 2024 May 9. doi: 10.3174/ajnr.A8174, 38548301 PMC11288544

[90] Matsukawa S Ishii A Fushimi Y Grinstead J Ahn S Kikuchi T Efficacy of high-resolution vessel wall MRI in the postoperative assessment of intracranial aneurysms following flow diversion treatment. J Neurosurg 2024;142:88–97. Published 2024 Aug 30. doi:doi: 10.3171/2024.5.JNS24174 39213673

[91] Jung SC Kim HS Choi CG Kim SJ Kwon SU Kang DW . Spontaneous and Unruptured chronic intracranial artery dissection: high-resolution magnetic resonance imaging findings. Clin Neuroradiol. (2018) 28:171–81. doi: 10.1007/s00062-016-0544-x, 27677627

[92] Zhu X Qiu H Hui FK Zhang Y Liu Y-e Man F . Practical value of three-dimensional high resolution magnetic resonance vessel wall imaging in identifying suspicious intracranial vertebrobasilar dissecting aneurysms. BMC Neurol. (2020) 20:199. doi: 10.1186/s12883-020-01779-0, 32434485 PMC7238595

[93] Hu L Quan K Shi Y Liu P Song J Tian Y . Association of Preoperative Vascular Wall Imaging Patterns and Surgical Outcomes in patients with Unruptured intracranial saccular aneurysms. Neurosurgery. (2023) 92:421–30. doi: 10.1227/neu.0000000000002219, 36637276

[94] Ma W Zhou K Lan B Chen K Li W Jiang G. Imaging investigation of cervicocranial artery dissection by using high resolution magnetic resonance VWI and MRA: qualitative and quantitative analysis at different stages. BMC Med Imaging. (2023) 23:184. doi: 10.1186/s12880-023-01133-z, 37957581 PMC10644659

[95] Cao Y Sun Y Yi Z Meng W Zhao X Feng X . Assessment of central nervous system vasculitis in children based on high-resolution vascular wall imaging. Rheumatol Adv Pract. (2024) 8:rkae038. doi: 10.1093/rap/rkae038, 38605731 PMC11009033

[96] Shimoyama T Uchino K Calabrese LH Hajj-Ali RA. Clinical characteristics, brain magnetic resonance imaging findings and diagnostic approach of the primary central nervous system vasculitis according to angiographic classification. Clin Exp Rheumatol. (2023) 41:800–11. doi: 10.55563/clinexprheumatol/a9886f, 37073640

[97] Patzig M Forbrig R Küpper C Eren O Saam T Kellert L . Diagnosis and follow-up evaluation of central nervous system vasculitis: an evaluation of vessel-wall MRI findings. J Neurol. (2022) 269:982–96. doi: 10.1007/s00415-021-10683-7, 34236502 PMC8264821

[98] Shimoyama T Uchino K Calabrese LH Hajj-Ali RA. Serial vessel wall enhancement pattern on high-resolution vessel wall magnetic resonance imaging and clinical implications in patients with central nervous system vasculitis. Clin Exp Rheumatol. (2022) 40:811–8. doi: 10.55563/clinexprheumatol/d3h5d6 35522543

[99] Sun LR Vossough A Kossorotoff M Bang OY Smith E Phi JH . Moyamoya across the lifespan: current neurologic care and future directions. Neurology. (2025) 104:e213484. doi: 10.1212/WNL.0000000000213484, 40036714

[100] Kim JY Kim HJ Choi EH Pan KH Chung JW Seo WK . Vessel Wall changes on serial high-resolution MRI and the use of Cilostazol in patients with adult-onset Moyamoya disease. J Clin Neurol. (2022) 18:610–8. doi: 10.3988/jcn.2022.18.6.610, 36367058 PMC9669557

[101] Yang H Huang G Li X Wu M Zhou W Yin X . High-resolution magnetic resonance vessel wall imaging provides new insights into Moyamoya disease. Front Neurosci. (2024) 18:1375645. Published 2024 Apr 11. doi: 10.3389/fnins.2024.1375645, 38665292 PMC11043609

[102] Ouyang F Wu Q Chen J Liu J Luo Z Yan M . Association of intravascular enhancement sign on 3D T1- weighted TSE sequences with cerebral perfusion and infarction events in moyamoya disease. Eur J Radiol. (2025) 190:112238. doi: 10.1016/j.ejrad.2025.112238, 40516503

[103] Lu M Zhang H Liu D Hao F Zhang L Peng P . Vessel wall enhancement as a predictor of arterial stenosis progression and poor outcomes in moyamoya disease. Eur Radiol. (2023) 33:2489–99. doi: 10.1007/s00330-022-09223-2, 36334103

[104] Ryu J Lee KM Woo HG Park JI Choi SK. Morphologic differences between ruptured and unruptured choroidal anastomosis in adult moyamoya disease: a high-resolution vessel wall imaging study. J Neurosurg. (2023) 140:441–9. doi: 10.3171/2023.6.JNS231017 37877970

[105] Eisenmenger LB Junn JC Cooke D Hetts S Zhu C Johnson KM . Presence of vessel wall hyperintensity in unruptured arteriovenous malformations on vessel wall magnetic resonance imaging: pilot study of AVM vessel wall "enhancement". Front Neurosci. (2021) 15:697432. doi: 10.3389/fnins.2021.697432, 34366779 PMC8334001

[106] McGuire LS Rizko M Brunozzi D Charbel FT Alaraj A. Vessel wall imaging and quantitative flow assessment in arteriovenous malformations: a feasibility study. Interv Neuroradiol. (2024) 30:694–701. doi: 10.1177/15910199221143189, 36471507 PMC11569463

[107] Stahl J McGuire LS Rizko M Saalfeld S Berg P Alaraj A. Are hemodynamics responsible for inflammatory changes in venous vessel walls? A quantitative study of wall-enhancing intracranial arteriovenous malformation draining veins. J Neurosurg. (2024) 141:333–42. doi: 10.3171/2024.1.JNS232625, 38552234

[108] Joo B Ahn SS Yoon PH Bae S Sohn B Lee YE . A deep learning algorithm may automate intracranial aneurysm detection on MR angiography with high diagnostic performance. Eur Radiol. (2020) 30:5785–93. doi: 10.1007/s00330-020-06966-8, 32474633

[109] Ryu WS Jeong S Park J Park D Kim H Lee M . Diagnostic accuracy of a deep learning algorithm for detecting Unruptured intracranial aneurysms in magnetic resonance angiography: a multicenter pivotal trial. World Neurosurg. (2025) 197:123882. doi: 10.1016/j.wneu.2025.123882, 40086726

[110] Li ZA Gao Y Ji K Zhang KY Zhang Q Wang J . AI-driven diagnosis of vulnerable intracranial atherosclerotic plaques using large language models and vision transformers: a multi-center study. Eur Radiol. doi: 10.1007/s00330-025-12065-3 41123642

[111] Lu M Zheng Y Liu S Zhang X Lv J Liu Y . Deep learning model for automated diagnosis of moyamoya disease based on magnetic resonance angiography. EClinicalMedicine. (2024) 77:102888. doi: 10.1016/j.eclinm.2024.102888, 39559186 PMC11570825

[112] Xu W Yang X Li Y Jiang G Jia S Gong Z . Deep learning-based automated detection of arterial Vessel Wall and plaque on magnetic resonance Vessel Wall images. Front Neurosci. (2022) 16:888814. Published 2022 Jun 1. doi: 10.3389/fnins.2022.888814, 35720719 PMC9198483

[113] Gao Y Li Z Zhai X Zhang G Zhang L Huang T . MRI-based habitat radiomics combined with vision transformer for identifying vulnerable intracranial atherosclerotic plaques and predicting stroke events: a multicenter, retrospective study. EClinicalMedicine. (2025) 82:103186. doi: 10.1016/j.eclinm.2025.103186, 40235946 PMC11999680

[114] Zhang J Wang W Dong J Yang X Bai S Tian J . Rapid vessel segmentation and reconstruction of head and neck angiograms from MR vessel wall images. NPJ Digit Med. (2025) 8:483. doi: 10.1038/s41746-025-01866-x, 40721485 PMC12304216

[115] Zhang X Sun W Zhang H Yang L Yang X Mao Y . Deep learning-based key point detection algorithm assisting vessel centerline extraction. Quant Imaging Med Surg. (2025) 15:4515–26. doi: 10.21037/qims-24-1949, 40384705 PMC12082607

[116] Yang J Xiao P Luo Y Zhu S Tang Y Chen H . Use of deep learning-based high-resolution magnetic resonance to identify intracranial and extracranial symptom-related plaques. Neuroscience. (2025) 571:130–8. doi: 10.1016/j.neuroscience.2025.02.055, 40032038

[117] Guo Y Akcicek EY Hippe DS HashemizadehKolowri SK Wang X Akcicek H . Long-term carotid plaque progression and the role of intraplaque hemorrhage: a deep learning-based analysis of longitudinal vessel wall imaging. J Cardiovasc Magn Reson. (2025) 28:102670. doi: 10.1016/j.jocmr.2025.102670, 41386425 PMC12808887

[118] Gao Y Li ZA Xie BC Wang WP Sun YC Wei ZQ . Deep learning network based on high-resolution magnetic resonance vessel wall imaging combined with attention mechanism for predicting stroke recurrence in patients with symptomatic intracranial atherosclerosis. Quant Imaging Med Surg. (2025) 15:2929–43. doi: 10.21037/qims-24-1723, 40235801 PMC11994518

[119] Ou C Li C Qian Y Duan CZ Si W Zhang X . Morphology-aware multi-source fusion-based intracranial aneurysms rupture prediction. Eur Radiol. (2022) 32:5633–41. doi: 10.1007/s00330-022-08608-7, 35182202

[120] Liu Y Yu Y Ouyang J Jiang B Yang G Ostmeier S . Functional outcome prediction in acute ischemic stroke using a fused imaging and clinical deep learning model. Stroke. (2023) 54:2316–27. doi: 10.1161/STROKEAHA.123.044072, 37485663 PMC11229702

[121] Li Z Huang T Zhang L Zhai X Zhao Q Lu H . A multi-view deep survival combined model for predicting stroke recurrence in symptomatic intracranial atherosclerosis. Acad Radiol. (2025) 32:S1076-6332(25)01032-3. doi: 10.1016/j.acra.2025.10.052 41242896

[122] Simon AB Hurt B Karunamuni R Kim GY Moiseenko V Olson S . Automated segmentation of multiparametric magnetic resonance images for cerebral AVM radiosurgery planning: a deep learning approach. Sci Rep. (2022) 12:786. doi: 10.1038/s41598-021-04466-3, 35039538 PMC8763944

[123] Peng F Xia J Zhang F Lu S Wang H Li J . Intracranial aneurysm instability prediction model based on 4D-flow MRI and HR-MRI. Neurotherapeutics. (2025) 22:e00505. doi: 10.1016/j.neurot.2024.e00505, 39617666 PMC11742858

[124] Tao X Shen S Yang L Chen K Yang H Mok GSP . Clinically oriented deep learning framework for automated vessel wall segmentation in black-blood MRI: a multi-center study. Eur Radiol. (2025) doi: 10.1007/s00330-025-12161-4 41273425

[125] Anwar SM Majid M Qayyum A Awais M Alnowami M Khan MK. Medical image analysis using convolutional neural networks: a review. J Med Syst. (2018) 42:226. doi: 10.1007/s10916-018-1088-1, 30298337

[126] Hilbert A Ramos LA van Os HJA Olabarriaga SD Tolhuisen ML Wermer MJH . Data-efficient deep learning of radiological image data for outcome prediction after endovascular treatment of patients with acute ischemic stroke. Comput Biol Med. (2019) 115:103516. doi: 10.1016/j.compbiomed.2019.103516, 31707199

[127] Clèrigues A Valverde S Bernal J Freixenet J Oliver A Lladó X. Acute and sub-acute stroke lesion segmentation from multimodal MRI. Comput Methods Prog Biomed. (2020) 194:105521. doi: 10.1016/j.cmpb.2020.105521, 32434099

[128] Guo Y Dou W Wang X Wang X Mao H Chen K. Can combined high-resolution vessel wall imaging and multiple post-labeling delay 3D pseudo-continuous arterial spin labeling differentiate moyamoya disease from atherosclerotic moyamoya syndrome? Eur J Radiol. (2023) 169:111184. doi: 10.1016/j.ejrad.2023.111184, 37931375

[129] Lu M Zhang H Liu S Liu D Peng P Hao F . Long-term outcomes of moyamoya disease versus atherosclerosis-associated moyamoya vasculopathy using high-resolution MR vessel wall imaging. J Neurol Neurosurg Psychiatry. (2023) 94:567–74. doi: 10.1136/jnnp-2022-330542, 36868848

